# Quantifying the Hardness of Bioactivity Prediction Tasks for Transfer Learning

**DOI:** 10.1021/acs.jcim.4c00160

**Published:** 2024-05-13

**Authors:** Hosein Fooladi, Steffen Hirte, Johannes Kirchmair

**Affiliations:** † Department of Pharmaceutical Sciences, Division of Pharmaceutical Chemistry, Faculty of Life Sciences, University of Vienna, Josef-Holaubek-Platz 2, 1090 Vienna, Austria; ‡ Christian Doppler Laboratory for Molecular Informatics in the Biosciences, Department for Pharmaceutical Sciences, University of Vienna, 1090 Vienna, Austria; § Vienna Doctoral School of Pharmaceutical, Nutritional and Sport Sciences (PhaNuSpo), University of Vienna, 1090 Vienna, Austria

## Abstract

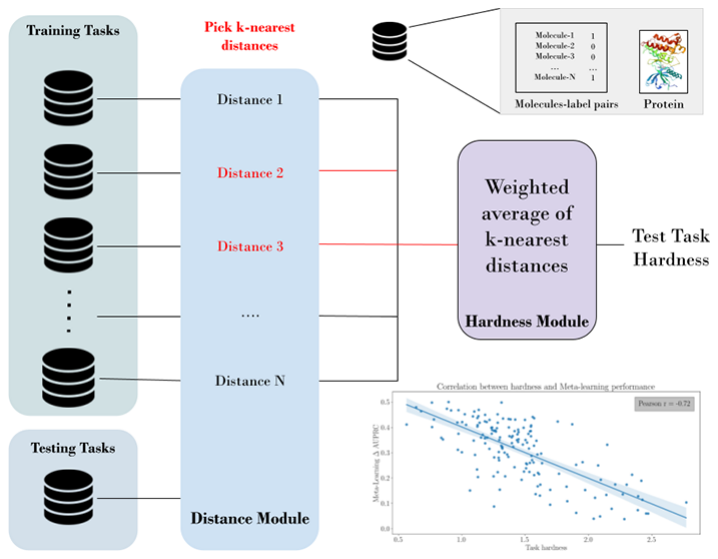

Today, machine learning methods are widely employed in drug discovery. However, the chronic lack of data continues to hamper their further development, validation, and application. Several modern strategies aim to mitigate the challenges associated with data scarcity by learning from data on related tasks. These knowledge-sharing approaches encompass transfer learning, multitask learning, and meta-learning. A key question remaining to be answered for these approaches is about the extent to which their performance can benefit from the relatedness of available source (training) tasks; in other words, how difficult (“hard”) a test task is to a model, given the available source tasks. This study introduces a new method for quantifying and predicting the hardness of a bioactivity prediction task based on its relation to the available training tasks. The approach involves the generation of protein and chemical representations and the calculation of distances between the bioactivity prediction task and the available training tasks. In the example of meta-learning on the FS-Mol data set, we demonstrate that the proposed task hardness metric is inversely correlated with performance (Pearson’s correlation coefficient *r* = −0.72). The metric will be useful in estimating the task-specific gain in performance that can be achieved through meta-learning.

## Introduction

In-silico methods for predicting the bioactivities or biomacromolecular targets of small organic compounds are of enormous importance to pharmaceutical, agrochemical, and cosmetics research. In recent years, machine learning has evolved into a key technology for bioactivity and target prediction.^1−4^ The limiting factor in further development of the computational methods is the measured bioactivity data available for model development.

The ChEMBL database^5,6^ is one of the most significant resources for measured small-molecule bioactivity data. It currently holds 2.4 Million compounds with more than 20 Million bioactivities recorded for a total of more than 15,000 targets. While this documents substantial scientific advances, these numbers are dwarfed by the size of the drug-like chemical space, which is estimated to comprise 10^23^ to 10^60^ compounds (for an overview, see Polishchuk et al.^7^), and the human proteome, which is composed of around 20,000 proteins.^8^ Aggravating data scarcity is the reciprocal relationship between novelty and data availability: for the most interesting biomolecular targets and the most innovative chemical space, data is, by definition, particularly scarce.^9−11^

Training machine learning models directly on small amounts of data can lead to overfitting, meaning that the resulting models will likely perform poorly under real-world scenarios (i.e., unseen, new tasks). Numerous knowledge-sharing methods have been devised and are increasingly used to address the challenges of the persistent data shortage.^12^ These include transfer learning,^13,14^ multitask learning,^15,16^ and meta-learning.^17−19^ Common to all knowledge-sharing methods is that they use information from related tasks (“source tasks” or “training tasks”) or data sets to improve the performance on tasks for which typically low and scarce amounts of data are available (“target tasks” or “test tasks”).

In transfer learning, the model is pretrained on “source tasks” (which ideally are related to the target task and for which ample data is available) and subsequently fine-tuned on the “target task”.^20,21^ By leveraging the knowledge gained from the source tasks, the model can learn the target task with smaller training data. In multitask learning, part of the model is usually shared between all the tasks (shared layers), and there are some specific layers on top for each task (task-specific layers). By sharing the parameters between the tasks, the aim is to achieve better performance with smaller-sized data sets and mitigate the risk of overfitting.^22,23^ In meta-learning, the goal is to learn common characteristics (for example, model initialization or representation learner) from training tasks and prior experience that can help the model adapt quickly (by observing a few positive/negative examples) to the target task.

It is widely recognized and intuitive that knowledge-sharing methods work best when the source and target tasks are similar (i.e., more relevant). Under some circumstances, knowledge-sharing methods may result in inferior models. For example, transfer learning may decrease performance on the target task (a phenomenon known as “negative transfer”^24^), e.g., if the source and target data distribution are too different.^25^ In multitask learning, it is well-known that naively adding source tasks can result in lower performance, and choosing a subset of similar tasks can mitigate the negative transfer problem.^26^ In the case of meta-learning, the performance improvement depends, among other factors, on the number of available training tasks and their relevance to the target task.^27,28^

Several methods have been proposed for quantifying the relatedness (i.e., distance or similarity) between tasks.^29−31^ For example, the discrepancy quantifies the distance between the source and target tasks. The measure relies on the loss function and hypothesis space of the predictive function.^32^ The utility of the discrepancy distance for establishing generalization bounds in domain adaptation (a subfield of transfer learning) depends on various parameters, including the complexity of the hypothesis class (often quantified using VC-dimension and Rademacher complexity). These complexities limit the applicability of the discrepancy distance in real-world scenarios.

Alternative methods for quantifying the distance between tasks can be applied more easily. Some of these methods rely on the model architecture for learning a task embedding (i.e., continuous representation of a task) and subsequently quantifying task relations (based on the distance in the continuous space such as Euclidean distance between tasks embedding). These models usually define the task embedding based on the parameters of an architecture (neural network) after training on each task. In particular, the Fisher information matrix of network parameters can be employed to capture the task structure (task embedding). An example of an architecture-dependent distance function is Task2Vec,^33^ which utilizes a probe network to quantify distance. The probe network can be any deep learning architecture (e.g., convolutional neural networks (CNNs) or graph neural networks (GNNs)) and is trained on all source and target tasks. The Fisher information matrix of the optimal parameters (i.e., the parameters after model training) is used to extract task representations and quantify task similarity. Using this metric, tasks that are semantically similar in the computer vision domain have been categorized in the same group. However, these methods for quantifying task relatedness have some drawbacks: They depend on the architecture of the probe network, and changing the architecture can alter the task embeddings and, consequently, the distance. Moreover, there is limited theoretical support for this distance concept.

Architecture-agnostic approaches rely solely on the feature-label pairs (*x* and *y*) present in the tasks (and not some auxiliary information like the weights of the optimal model). For example, one of the most compelling architecture-agnostic approaches, the optimal transport distance for data sets (OTDD),^34^ does not require training of the network on multiple tasks (or data sets). It has a theoretical guarantee and explanation since it originates from optimal transport theory and can also be applied if the labels between two data sets are disjointed. However, the computational cost of this method is high, limiting its applicability. To increase computational efficiency, entropy regularization has been introduced.^35^ Entropy regularization creates a trade-off between computational cost and accuracy.

While several methods for quantifying task distance have been devised, the concept of task distance has yet to be thoroughly explored in certain scientific domains, including areas related to small molecule research and, more specifically, bioactivity prediction. In this context, a relevant question is how much information available about small molecules interacting with a protein (e.g., measured bioactivities) can be transferred to another protein. Answering this question requires reliable measures of the distance between tasks, which include biomacromolecular targets and bioactivity (drug–target interaction) data sets.

To address the challenge of data scarcity in molecular property prediction tasks, Han Li et al.^36^ proposed MoTSE, a task similarity-enhanced transfer learning strategy designed to enhance molecular property prediction performance on data sets with limited labels. MoTSE involves pretrained a graph neural network on each task and feeding probe data sets into each pretrained network. Subsequently, attribution-based and molecular representation similarity analyses are applied to define the distance between the tasks. The most similar source task is then utilized to pretrain a network, which is subsequently fine-tuned on the target task. While the results reported for MoTSE are encouraging, it is worth noting that the method for defining distance is sensitive to both the choice of GNN architecture and the selection of the probe data set.

Huang et al.^37^ proposed a method for grouping auxiliary molecular data sets to enhance performance on the target data set. They utilized the Task2Vec method for determining the distance between tasks and subsequently used this distance, in combination with structural distance, for grouping auxiliary tasks. Despite being a promising approach, the reported correlation between the relative improvement in performance and task distance is not substantial. Additionally, Task2Vec is an architecture-dependent distance, meaning that the defined distance is sensitive to the probe network. Moreover, this work did not specifically focus on bioactivity prediction and, as a result, did not consider information related to the protein target for defining the task distance.

**Figure 1:**
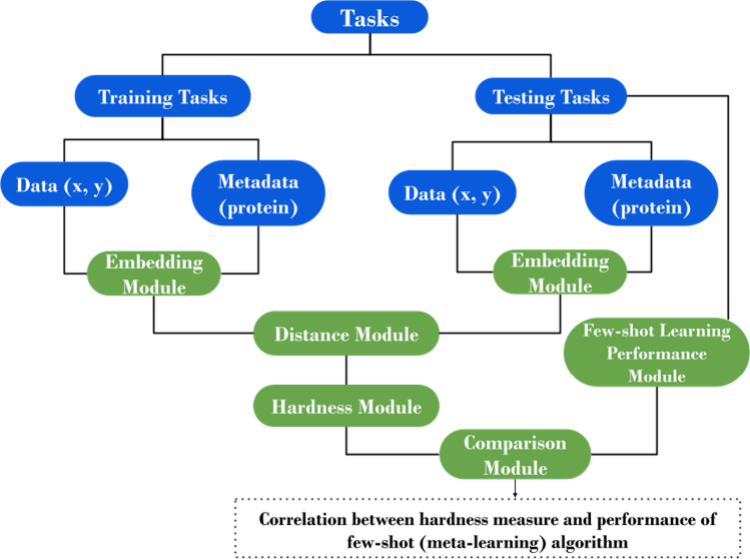
General overview of the workflow followed in this work. First, we determine the embedding for both data (i.e., molecules and bioactivity label pairs) and metadata (i.e., proteins). In the next step, we determine the distance between all of the training and test tasks and the hardness, which includes both external and internal hardness for each test task (external hardness is the average of the *k*-nearest distance to the training tasks). Finally, to verify our method, we calculate the correlation between the test task’s hardness and improvement gain on each test task using a meta-learning method.

Here, we present the development and evaluation of a metric designed to quantify task hardness, i.e., the difficulty level posed by a test task conditioned on the available source tasks, in the context of bioactivity prediction (see Figure 1 for an overview of the complete workflow). Each task comprises a set of known active and inactive small molecules (representing the chemical space) and a protein target (representing the protein space). The task hardness metric comprises three components:

1 External chemical space hardness (EXT_CHEM): quantifies the relatedness (distance) of the chemical space between the source task and the target tasks.
2 External protein space hardness (EXT_PROT): quantifies the relatedness (distance) of the source protein and the protein space of the target tasks.
3 Internal chemical space hardness (INT_CHEM): quantifies the difficulty of the target task based on the distribution of chemical space in the training and validation set of the target task.

Together, the external hardness components quantify the extent of relevant information available in the source tasks to aid in solving the target task.

To calculate EXT_CHEM, we employ desc2D,^38^ ChemBERTa-77M-MLM,^39^ Uni-Mol,^40^ Gin supervised contextpred, Gin supervised edgepred, Gin supervised masking, Gin supervised infomax,^41^ and Roberta-Zinc480M-102 M methods to produce representations of molecules from their SMILES notation. We then compute a distance matrix of the test task to the source tasks using an architecture-agnostic approach (optimal transport data set distance, OTDD). We finally calculate the EXT_CHEM as the average (or weighted average) distance to the *k*-nearest source tasks from this distance matrix.

To determine EXT_PROT we follow the same logic. First, we employ the evolutionary scale modeling (ESM-2) approach^42,43^ to generate a representation of proteins from their sequences. We then compute a distance matrix based on the Euclidean distance of the test task from the source tasks. We finally calculate the EXT_PROT from this distance matrix as the average distance to the *k*-nearest source tasks.

To calculate the INT_CHEM, we partitioned the test task into training and validation sets. Subsequently, we train a random forest or *k*-nearest neighbor (kNN) model on the training set and measure the Area Under the Receiver Operating Characteristic Curve (ROC-AUC) on the validation set. Finally, we use (1-ROC-AUC) as the measure of INT_CHEM.

We validate our proposed task hardness metric by comparing the assigned hardness level to test tasks with the observed performance change after applying meta-learning. This process helps affirm our metric’s effectiveness in assessing task hardness. Subsequently, we demonstrate the practical utility of this concept when applying a meta-learning algorithm to enhance the performance of the target task by using available source tasks.

The FS-Mol^11^ data set serves as the foundation for model development and validation. It is a comprehensive data set derived from the ChEMBL database, designed for benchmarking machine learning models for bioactivity prediction. The data set is particularly suitable for testing knowledge sharing approaches because of the large number of training tasks (4938 tasks) and the substantial number of disjoint test tasks (157 tasks). The individual tasks are nearly balanced, meaning the number of active to inactive samples is similar for most tasks (Figure S1). The source tasks represent mostly a single protein assay related to an enzyme target (Figure S2). However, among the source tasks are some assays (7%) representing more than one protein and assays with no protein family specified (33%). Most source tasks comprise fewer than 400 molecules (Figure S3). Each of the 157 test tasks represents a single-protein assay related to an enzyme target (Figure S2). Most test tasks comprise fewer than 1000 (Figure S3).

## Materials and Methods

### Data Set

The unmodified FS-Mol^11^ served as a data set for method development and evaluation. It comprises 4938 training and 157 test tasks.

### Quantifying Task Hardness

The workflow for quantifying the hardness of a bioactivity task (Figure 1) comprises the following modules: embedding, distance, hardness, and few-shot learning performance. In this section, we describe each of these modules and provide explanations of their functioning.

Each task contains a pair of data and metadata, , where data contains a set of measured active or inactive compounds against a particular protein target *D* = {(*x*_*i*_, *y*_*i*_)}_*i*=1_^*n*^, and metadata is a protein sequence obtained from UniProt^44^ using UniProt IDs. In particular, we extracted target ChEMBL IDs from ChEMBL assay IDs, determined UniProt IDs, and finally retrieved the protein sequence from the UniProt database.

**Figure 2:**
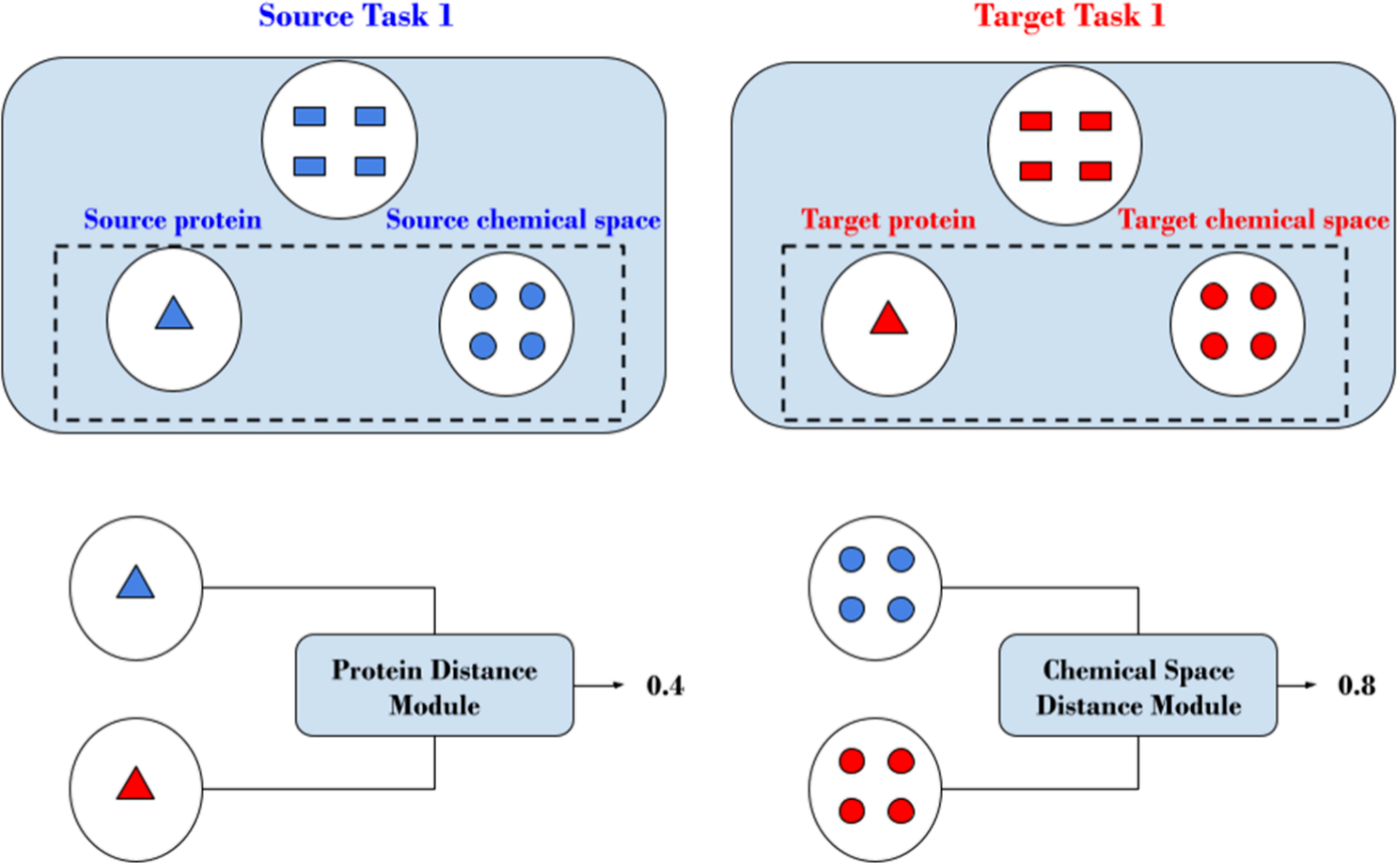
Overview of the distance module. Distances are calculated for each pair of training and test tasks for both proteins (metadata) and chemical space (molecule-label pairs). The result of the distance module is matrices representing the source tasks (rows) and target tasks (columns).

We quantify the hardness of a bioactivity prediction task based on three components: EXT_CHEM, EXT_PROT, and INT_CHEM. The two external components define the hardness of a target task based on the source tasks (i.e., the distance between the source tasks and the test task). The chemical space hardness is defined by the distance between the chemical space of the small molecules of the source and target tasks (Figure 2). Likewise, the protein space hardness is defined by the distance between the representations of the proteins of the source and target tasks (Figure 2). The internal component is solely a function of the target task and does not access any information related to the source tasks. It measures the inherent difficulty of the task based on machine learning performance on the internal train and test split without considering external information.

Each component of the hardness module (i.e., EXT_CHEM, EXT_PROT, and INT_CHEM) captures a distinct facet of task hardness and contains some independent information. Therefore, combining these components is expected to lead to a more accurate measure of hardness, where “accuracy” is defined in terms of its inverse correlation with the result or performance of the meta-learning (prototypical network).

The hardness of a bioactivity prediction task results from combining the individual components:
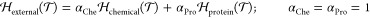
1
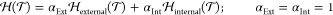
2

In this work, we assign equal weights to all of the defined hardness modules for simplicity. We normalize each component separately to the [0–1] range and then add them together. However, users can assign weights based on their preferences and data availability. For instance, if a target task’s train or validation split is unavailable, the user can assign zero weight to the internal hardness. A higher weight can also be assigned to the chemical space component if users want to prioritize the chemical space hardness.

### External Chemical Space Hardness (EXT_CHEM)

Each task comprises known active and known inactive small molecules (*x*) and the corresponding bioactivity labels for each molecule (*y*). Given {(*x*_*i*_^*A*^,*y*_*i*_^*A*^)}_*i*=1_^*n*^ for the first data set *D*_*A*_ and {(*x*_*i*_^*B*^,*y*_*i*_^*B*^)}_*i*=1_^*m*^ for the second data set *D*_*B*_, this part aims to quantify the similarity between *D*_*A*_ and *D*_*B*_ from a chemical space perspective. The distance between the data sets is the distance between two sets of labeled molecules.

Quantifying EXT_CHEM involves the following steps:

1 Generation of a molecular representation (continuous embedding) for each data set and, specifically, each molecule in the individual data set, based on different methods.
2 Calculation of the distance matrices between pairs of data sets with the OTDD.
3 Determination of the hardness of each test task based on the average (or weighted average) distance between the test task and the *k*-nearest source tasks.

The following featurizers for calculating molecular representations were explored in this study:

- Set of 215 2D physio-chemical descriptors implemented in RDKit^38^ (based on the “molfeat” package implementation). The molfeat implementation has six additional features compared to the RDKit descriptors. These features include the number of atom stereocenters, number of unspecified atom stereocenters, number of bridgehead atoms, number of amid bonds, number of spiro atoms, and number of structural alerts present in a molecule (based on the structural alerts defined in the RDKit Quantitative Estimate of Druglikeness -QED-^45^ score package).
- ChemBERTa-77M-MTR and ChemBERTa-77M-MLM:^39^ ChemBERTa is a pretrained language model for molecules based on (Ro)BERT(a)^46^ and trained on around 77 M PubChem compounds. MLM is a masked language model, where the model has been pretrained to predict and identify the masked token (SMILES character) from the context. MTR is a multitask regression model, which has been pretrained by predicting 200 molecular properties from SMILES in a multitask manner.
- Uni-Mol:^40^ Uni-Mol is a model based on the 3D representation of a molecule. The model has been pretrained on 209 M molecules based on the 3D position recovery and masked atom prediction.
- Gin supervised contextpred, Gin supervised edgepred, Gin supervised masking, and Gin supervised infomax:^41^ These models are based on pretraining a graph isomorphic network (GIN)^47^ on molecular data. The models have been trained on around 2 million graphs from the ChEMBL database, encompassing both graph-level and node-level prediction tasks. Graph-level pretraining is achieved through supervised learning, where the GIN is pretrained to predict multiple molecular properties from the input graph. Node-level pretraining has been conducted using context prediction or atom (or edge) mask prediction. Additionally, Gin supervised infomax is pretrained with the Infomax approach,^48^ a method for training node encoders to maximize the mutual information between local nodes and the entire graph representation.
- Roberta-Zinc480M-102M: This masked language model has been pretrained on around 480m SMILES strings from the ZINC database. The model is a transformer with 102 M parameters pretrained by predicting the masked token (i.e., SMILES string characters) from the context.

The Uni-Mol features were calculated using code from the Uni-Mol GitHub repository.^40^ All other features were calculated with the “molfeat” Python package. A detailed description of each feature calculation method is provided in the Supporting Information.

After determining features for all of the molecules in the source and target tasks, we calculate the distance between the chemical space of the source and target tasks using the OTDD approach.
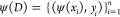
3
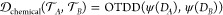
4

OTDD is a method for calculating the distance between two labeled data sets based on optimal transport theory.
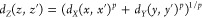
5where *z* = (*x*,*y*) is a molecule-label pair and distance is defined between features (*x*, *x*′) and also between labels (*y*, *y*′). The distance between the features is the Euclidean distance. The following distribution is considered for labels:
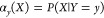
6With this definition, the distance between labels is transformed to the distance between their associated distributions. Again, optimal transport can be used to define the distance between these distributions, which can lead to the *p*-Wasserstein distance between labels and, consequently, the following distance between feature-label pairs:
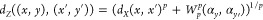
7

If the distribution of α_*y*_ is considered as a Gaussian distribution, the 2-Wasserstein distance between the Gaussian distribution has an analytical form, which depends on the mean and covariance of the two distributions.
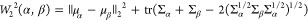
8

Hence, this OTDD distance has been considered to quantify the distance between molecules and labels in our source and target tasks.

At this stage, a list of numbers representing the distance of the target task from all of the source tasks has been generated for each target task. However, we aim to assign a single number to each target task that quantifies its difficulty, considering all of the source tasks from a chemical space perspective. The intuition is that after applying meta-learning, we can also derive a single number for each task indicating the improvement or decline in prediction performance, leveraging all the information from the source. Various aggregation functions could be employed to distill the list into a single number. We opted for the average of the *k*-nearest data set distance as our aggregation method. We consider the k closest source tasks for a given target task and average their distances to calculate the task hardness. This process is repeated for all of the target tasks. If *k* source tasks are close to the test task (based on the defined metric), then the hardness would be small, and the task would be considered easy. The opposite is true when the task is considered to be hard. Ultimately, we normalize these hardness numbers using the Min-Max normalization schema.
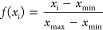
9
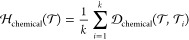
10

**Chart 1:**
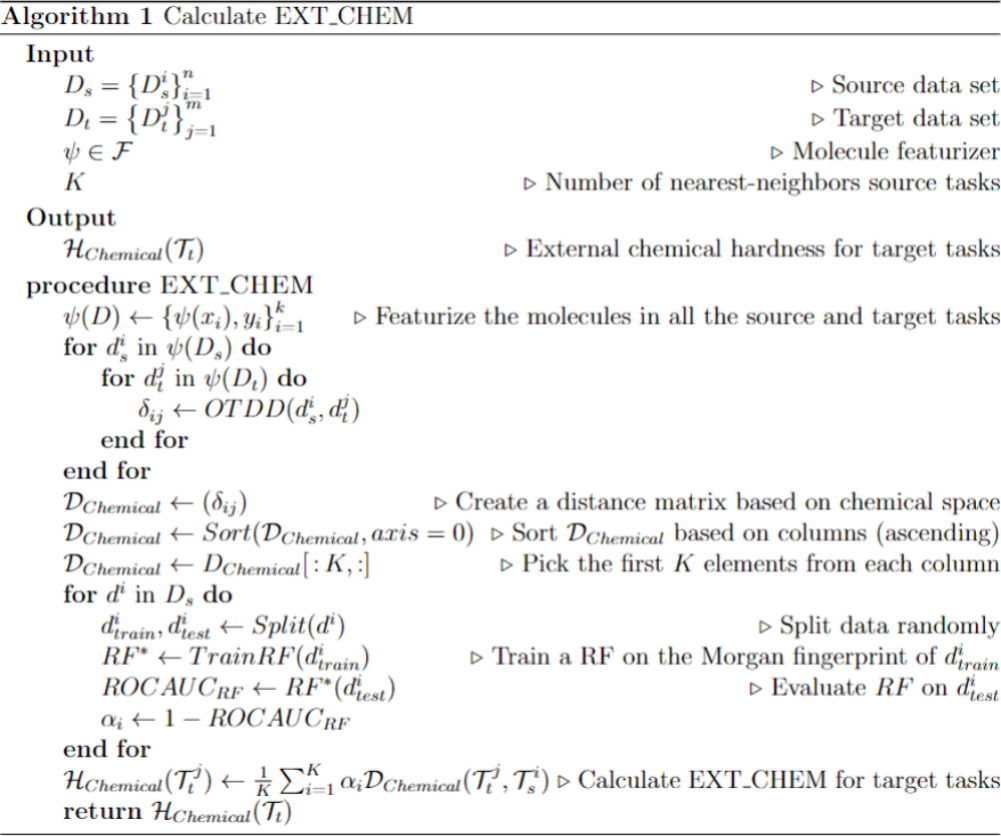


Rather than simple averaging, we implemented weighted averaging to determine EXT_CHEM. The underlying intuition is that even with numerous similar tasks in the training set, if all of them are challenging for a single-task machine learning method, the shared information may not significantly support the ability to solve the test task. The weights for calculating the average distance are derived from the INT_CHEM of each training task. Consequently, we first define a difficulty measure for each training task, calculated as the complement of the ROC AUC of the random forest (RF) validation performance, using only 16 data points for training. These difficulty measures are then employed as weights assigned to each source task for averaging. Further details can be found in Algorithm 1 (Chart 1).
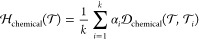
11

### External Protein Space Hardness (EXT_PROT)

Each task contains information about proteins. We refer to this data type as metadata, because it is not used in training any machine-learning model (including the prototypical networks employed in meta-learning). Since protein structural information is absent for many proteins, we use protein sequence data to represent proteins and compute distances. Various machine learning methods for learning a representation of a protein based on the sequence have been reported.^49−51^ In this work, we employ evolutionary scale modeling (ESM2) to learn a representation of the proteins.^42,43^ The mean representation for each token (amino acid residue) was employed as the final protein representation. The architecture of the ESM2 model is a transformer pretrained with the masked language modeling task.^14^ Several models and setups were explored (Table 1).

**Table 1:** ESM2 Models Explored for Extracting Protein Representations

Model name	Number of layers	Number of model parameters	Representation aggregation method
ESM2_t6_8M_UR50D	6	8M	Mean
ESM2_t12_35M_UR50D	12	35M	Mean
ESM2_t30_150M_UR50D	30	150M	Mean
ESM2_t33_650M_UR50D	33	650M	Mean
ESM2_t36_3B_UR50D	36	3B	Mean

**Figure 3:**
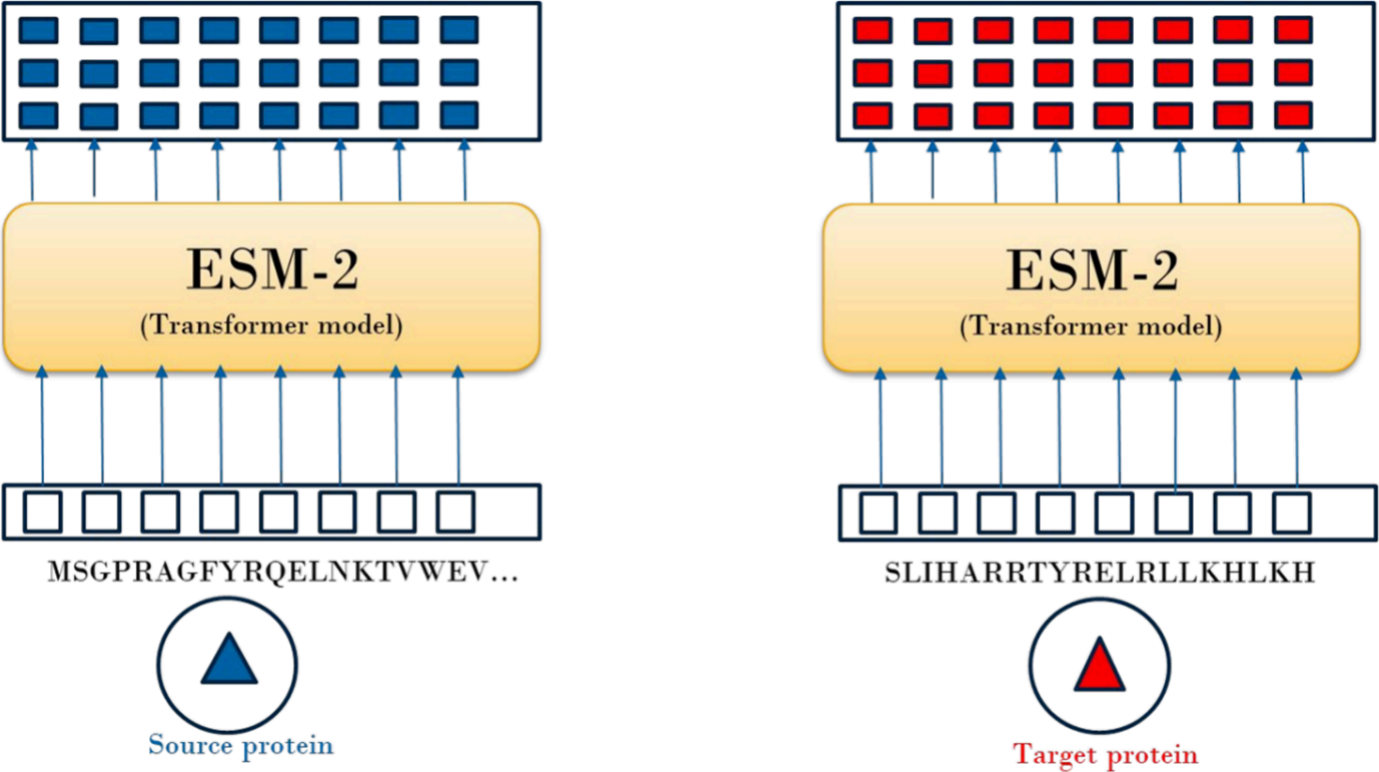
Schematic overview of the process of determining protein representations for source and target tasks. Protein sequences (amino acid residues) are fed into the ESM2 models, which return representation for each token or amino acid residue. Subsequently, the final protein representation is obtained through mean aggregation over all tokens.

The presence of proteins in the source tasks similar to the protein of the test task can make a target task easier from a protein perspective (because knowledge can be transferred from the training tasks to the test task). We executed the following systematic procedure for all tasks containing just a single protein:

1. Embed or encode a protein in continuous space (Figure 3).
2. Define a distance metric (Euclidean or cosine) in this embedded space.
3. Calculate hardness based on the distance matrices.

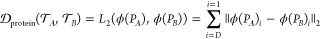
12
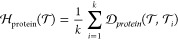
13

Approximately 7% of the training tasks (and no test tasks) represent protein complexes (i.e., more than one protein). For these tasks, we calculated the distance of the target protein with all of the proteins in the complex and selected the largest distance.

**Chart 2:**
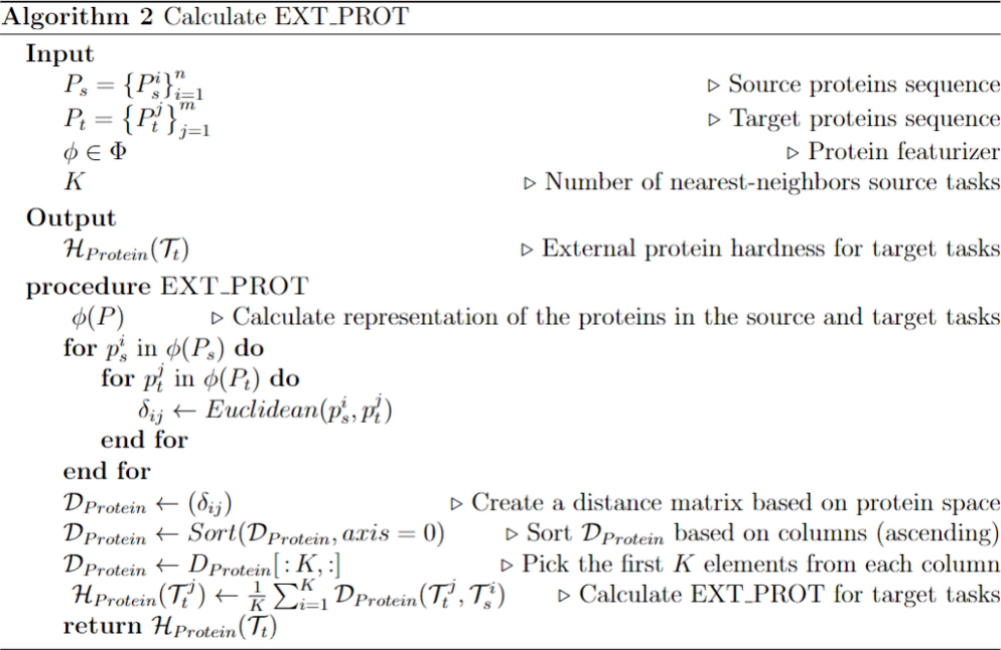


The result of the calculation of the distances between the proteins in the test and training tasks is a *N*_train_ × *N*_test_ matrix (*D*_*N*_train_ × *N*_test__). EXT_PROT is determined for each task based on this matrix. The output is a *N*_test_ dimensional vector in which each element indicates the EXT_PROT of a particular task. For this purpose, we utilize the average of *k*-nearest source tasks to assign a hardness value to each test task. Further details can be found in Algorithm 2 (Chart 2). Additional information on determining protein representations for all source and target tasks is provided in the Supporting Information.

### Internal Chemical Space Hardness (INT_CHEM)

Each task contains active and inactive compounds (*x*) with corresponding bioactivity labels (*y*). The structural relationship between the active and inactive compounds can define the hardness of a task. Also, the splitting strategy employed in defining training and test sets can have a decisive effect on task hardness.

Random splitting, which assigns the data randomly into training and test sets, can be considered easier for the machine learning methods than scaffold splitting, which splits the data in the training and test set based on different scaffolds.^52,53^ In this study, we use (1-ROC-AUC) on a validation set of single-task machine learning methods (including random forest and kNN) trained on each test task as the measure for internal task hardness.

**Chart 3:**
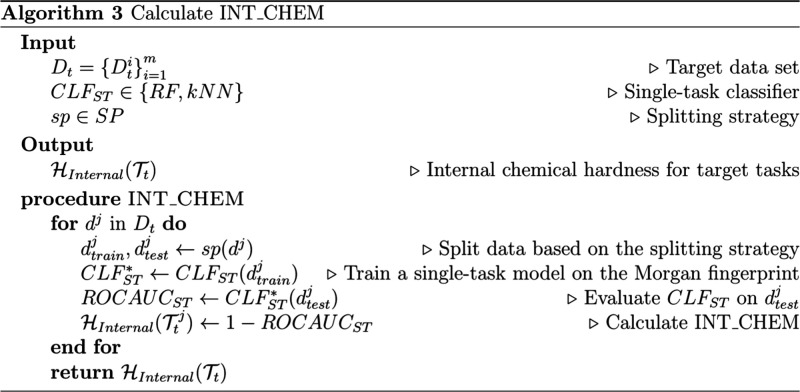


We employed the following methods for calculating INT_CHEM:

- Random forest with *m*_train_ training samples (*m*_train_ = 16, 32, 64)
- kNN with *m*_train_ training samples (*m*_train_ = 16, 32, 64)

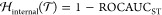
14Further details can be found in Algorithm 3 (Chart 3).

In this work, we performed stratified random splitting of individual target tasks to determine the Pearson correlation coefficient r for INT_CHEM and the prototypical network performance based on ROC-AUC. In addition, we performed experiments with other splitting strategies (scaffold and random splitting).

If a single-task method achieves a ROC-AUC of close to 1, the internal task hardness would be near 0 (i.e., the task would be considered easy internally), and vice versa.

It is important to note that this metric captures hardness from an orthogonal (complementary) perspective with respect to external task hardness. External task hardness is sensitive to the availability of similar source tasks, whereas INT_CHEM focuses on the isolated test task. INT_CHEM is sensitive to the relationship between the active and inactive compounds and the data-splitting method.

### Comparison with Meta-Learning Performance

**Chart 4:**
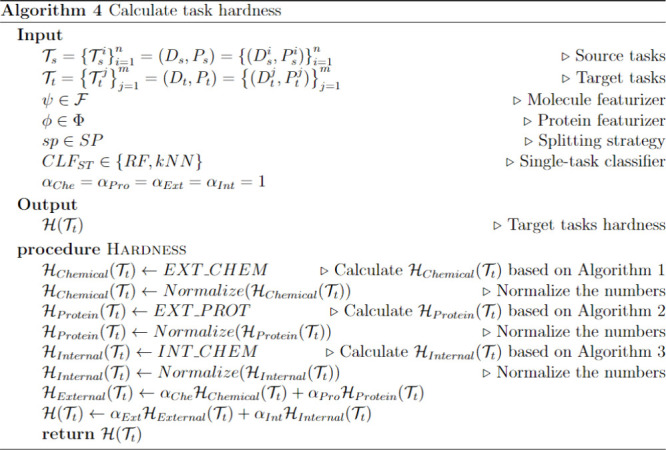


In this study, meta-learning serves as a method to validate the proposed task hardness metric. For each test task, we define a level of task hardness based on Algorithm 4 (Chart 4). Additionally, we observe the extent to which the performance (e.g., ROC-AUC or AUPRC) improves through meta-learning. Subsequently, we compare these two metrics to determine whether an inverse correlation exists between task hardness and performance improvement from meta-learning. We anticipate that tasks with higher hardness values will benefit less from meta-learning.

The goal of meta-learning is to distill knowledge from training tasks (such as the initialization of a neural network or representation learner network) and apply this knowledge to the distribution of unseen test tasks. This enables meta-learning to adjust to test tasks efficiently, even when only a few samples are available.^54^
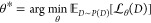
15

The aim is to identify the parameters that minimize the loss function over the distribution of tasks. In this formulation, one task and the corresponding data set are considered as one data point sampled from the distribution over tasks.

Depending on the nature of this knowledge and how a meta-learner utilizes this information on a new task, two types of meta-learning can be distinguished: optimization-based and embedding-based meta-learning. In optimization-based meta-learning, the model is trained to adapt quickly to a new task with a small number of training samples. Model agnostic meta-learning (MAML) is a prominent example of the optimization-based method, where the experience is an initialization of the neural network learned from a distribution of related tasks.^18^ With the appropriate initialization, the model can quickly learn a new unseen task based on only a few examples by gradient descent.
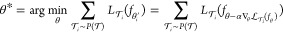
16

Embedding-based methods learn a new metric space based on the distribution of related tasks. Providing just a few labeled examples in this learned metric space is sufficient to achieve a good machine-learning performance. A prominent example of embedding-based approaches is prototypical networks. Prototypical networks classify samples based on the distance between each class prototype in the learned metric space.^19^ For a test task, the prototypical network does not require further optimization or gradient-based training. Instead, it calculates the representation of class prototypes and then assigns a class probability to new samples based on the distance from the prototype. Because the prototypical network does not need further optimization, it is faster for new tasks than optimization-based methods. Prototypical networks have been shown to perform at least as well or better than other meta-learning methods^54^ for few-shot classification.
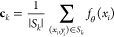
17
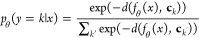
18

In this formulation, *S*_*k*_ denotes the sample set from class *k* in the support set (for just two classes, *k* can be 0 or 1). *c*_*k*_ represents the prototype representation of class *k*, which is the average of the representation of the samples in class *k*.

After the prototype for the new example (*x*) in the query set is determined, the probability of the label is assigned based on the distance of the sample from all the prototypes.

**Chart 5:**
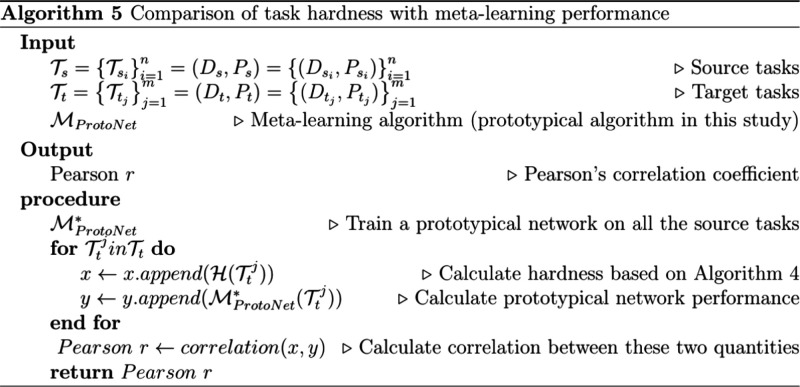


In this study, we employ the prototypical network implemented in the FS-Mol package and train each test task using 128 samples (support size) (see the Supporting Information for details on the network architecture and hyperparameters). We compare the improvement or decline in the performance of prototypical networks to the hardness assigned to each test task. This analysis aims to explore the correlation between these two metrics. Further details can be found in Algorithm 5 (Chart 5).

### Selection of Relevant Source Tasks for a Specific Target Task

The distance and hardness metric introduced in this work can also aid in selecting relevant source tasks for knowledge transfer and meta-learning. In essence, given a target task and a list of all available source tasks, the most relevant source tasks can be identified based on their chemical and protein space distance from the target task, and transfer learning can be applied based on this informed selection.

**Chart 6:**
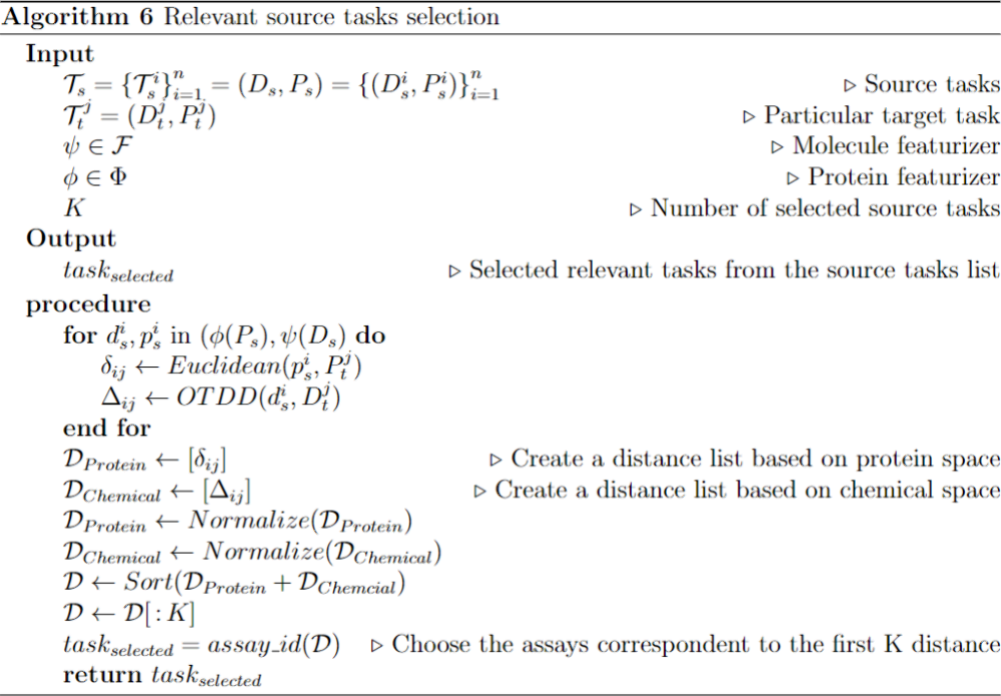


In this study, for each target task, we select the most relevant source tasks based on the external chemical and protein space distance metrics. Subsequently, we train a prototypical network on the selected list and assess its performance on the target task. In addition, we determined the benefit of the rational source task selection over a random selection of source tasks. Further details about task selection can be found in Algorithm 6 (Chart 6).

## Results and Discussion

We first compare the performance of a random forest model (as an example of a single-task method) with that of a prototypical network (as an example of a method that utilizes information from all source tasks) and demonstrate that the latter achieves better results for the majority of 157 test tasks. Subsequently, we reveal instances (i.e., test tasks) where tasks exhibit similar performance in the random forest but markedly different performance in the prototypical network. These examples underscore that some test tasks benefit more from sharing knowledge than others and stimulated experiments quantifying task hardness with our proposed method. Following these experiments, we conducted ablation studies to determine the sensitivity of EXT_CHEM and EXT_PROT to different representations used for molecules and proteins.

### Performance of Single-Task Learning versus Meta-Learning as a Function of the Support Set Size

**Figure 4:**
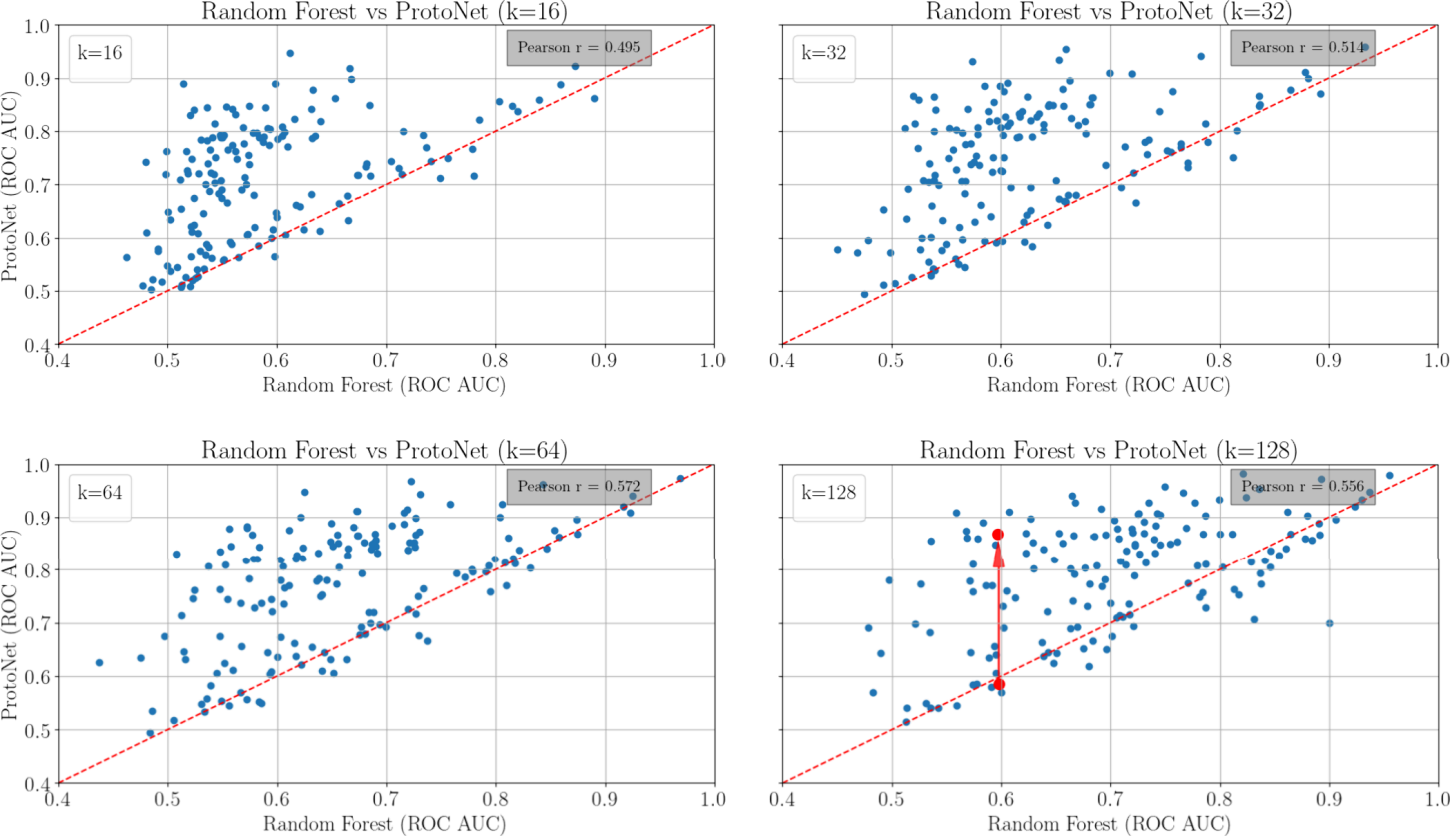
Comparison of the performance of the prototypical network with the random forest approach on 157 test tasks of the FS-Mol benchmark data set. We use the (1-ROC-AUC) of the random forest approach to measure internal task hardness.

When using support sets composed of just 16 randomly selected training samples (i.e., labeled molecules) for each of the 157 test tasks of the FS-Mol benchmark set, the prototypical network outperformed the random forest in 140 of the tasks (average ROC-AUC 0.70 vs 0.58, representing a plus of 0.120; Figure 4). As the size of the support sets was increased, the advantage of the prototypical network over the random forest diminished. Measured as ΔROC-AUC, the benefit of the meta-learning approach over the single-task approach decreased from 0.120 for *n*_train_ = 16 (and *n*_train_ = 32) training samples to +0.092 for *n*_train_ = 128. These results confirm that meta-learning yields better results in low-data regimes.

More informative than the performance averages is a look at how the two methodologically distinct approaches perform in the individual tasks. For runs with a support set size of 16 samples, the Pearson’s correlation coefficient *r* for the ROC-AUC values was 0.495. With larger support set sizes, the Pearson’s *r* increased slightly, up to *r* = 0.555 for *n*_train_ = 128. These results indicate a moderate correlation between the performance of the single-task and meta-learning approaches.

As apparent from the scatter plots presented in Figure 4, a subset of tasks poses a similar hardness level to the prototypical network and the random forest (i.e., those dots placed in proximity of the diagonal), whereas other tasks are perceived as being more challenging by either method. Interestingly, large spreads in ROC-AUC values are observed for the prototypical network. For example, for tasks for which the random forest reaches ROC-AUC values of around 0.60, the values for the prototypical network range from 0.57 to 0.86 (indicated in Figure 4 by the red dots and arrows). This means that many tasks pose a comparable hardness to the random forest (similar internal hardness) but a distinct hardness to the prototypical network (distinct external hardness).

Both internal and external hardnesses influence the absolute performance of the prototypical network. The prototypical network’s performance can be decomposed into two components: the one associated with INT_CHEM (conceptually represented by the performance of the single-task random forest approach) and the performance improvement resulting from knowledge transfer associated with EXT_CHEM and EXT_PROT.

**Figure 5:**
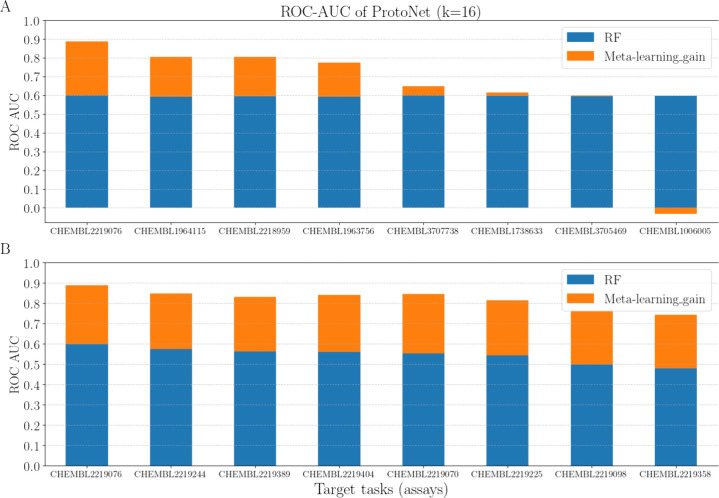
Performance of the meta-learning approach (prototypical network) dissected into a component associated with INT_CHEM (conceptually represented by the performance of the single-task random forest approach; blue) and a component associated with the performance improvement resulting from knowledge transfer associated with EXT_CHEM and EXT_PROT (orange). (A) Representation of all target tasks for which the random forest obtained ROC-AUC values of approximately 0.6; (B) representation of all target tasks for which the gains associated with knowledge-sharing were comparable (ROC-AUC values +0.26 to +0.29).

Figure 5A presents the test tasks for which the random forest obtained ROC-AUC values of approximately 0.60 (± 0.001), indicating a comparable internal hardness. However, the gains in performance associated with meta-learning vary significantly, ranging from +0.29 to −0.03 in the ROC-AUC values.

Figure 5B presents the test tasks that show similar gains associated with meta-learning (ROC-AUC values of +0.26 to +0.29), indicating a comparable external hardness. However, they exhibit different internal hardness levels, represented by ROC-AUC values of the random forest models between +0.47 and +0.60.

The disparity and gaps in the improvements gained from knowledge transfer (visualized by the orange bars in Figure 5) could be a result of differences in the relevance of the source tasks to the individual test tasks. It is intuitive to hypothesize that the prototypical network performs particularly well on test tasks sufficiently closely related to the source tasks so that knowledge can be transferred. Understanding these relationships is highly important because it could enable (i) the task-specific prediction of the benefit of meta-learning approaches and (ii) performance optimization of meta-learning approaches by enabling the selective utilization of source tasks.

The data splitting strategy (stratified random, random, and scaffold splitting) can potentially influence the internal hardness. However, the observed variations related to the employed splitting strategy were minor (Table S4) because of the balanced representation of active and inactive compounds in most target tasks (Figure S1) and the high scaffold diversity (Figure S11).

### Task Hardness and Its Relation to Meta-Learning Performance in a Defined Modeling Setup

We define task hardness as a measure of the difficulty a test task poses to a machine learning model for bioactivity prediction. Our definition of task hardness is defined by three components: EXT_CHEM (quantified as the weighted average OTDD distances between the molecule representations of the test task and the *k*-nearest training tasks), EXT_PROT (quantified as the average of the Euclidean distances between the protein representations of the test task and the *k*-nearest training tasks) and INT_CHEM (quantified as 1-ROC-AUC of a random forest classifier trained on randomly selected 16 samples and tested on the remaining samples in the test task, see Figure 4). Full details are provided in the Methods section.

We determined the characteristics of the hardness components based on the 157 test tasks of the FS-Mol benchmark. Multiple modeling setups and parameters were explored to determine the method’s sensitivity. These will be discussed in subsequent sections of this work. Here, we begin our analysis using one defined modeling setup: representation of the small molecules with GIN-supervised infomax; representation of the proteins with ESM2-t33–650M; consideration of the ten nearest source tasks (accounting for approximately 0.2% of the source tasks) for calculating hardness from distance matrices. Normalization was applied to have all values fall within the range of 0.0 to 1.0. This default modeling setup is representative of all explored setups, and as we will see later, the conclusions drawn from any explored modeling setups are consistent.

**Figure 6:**
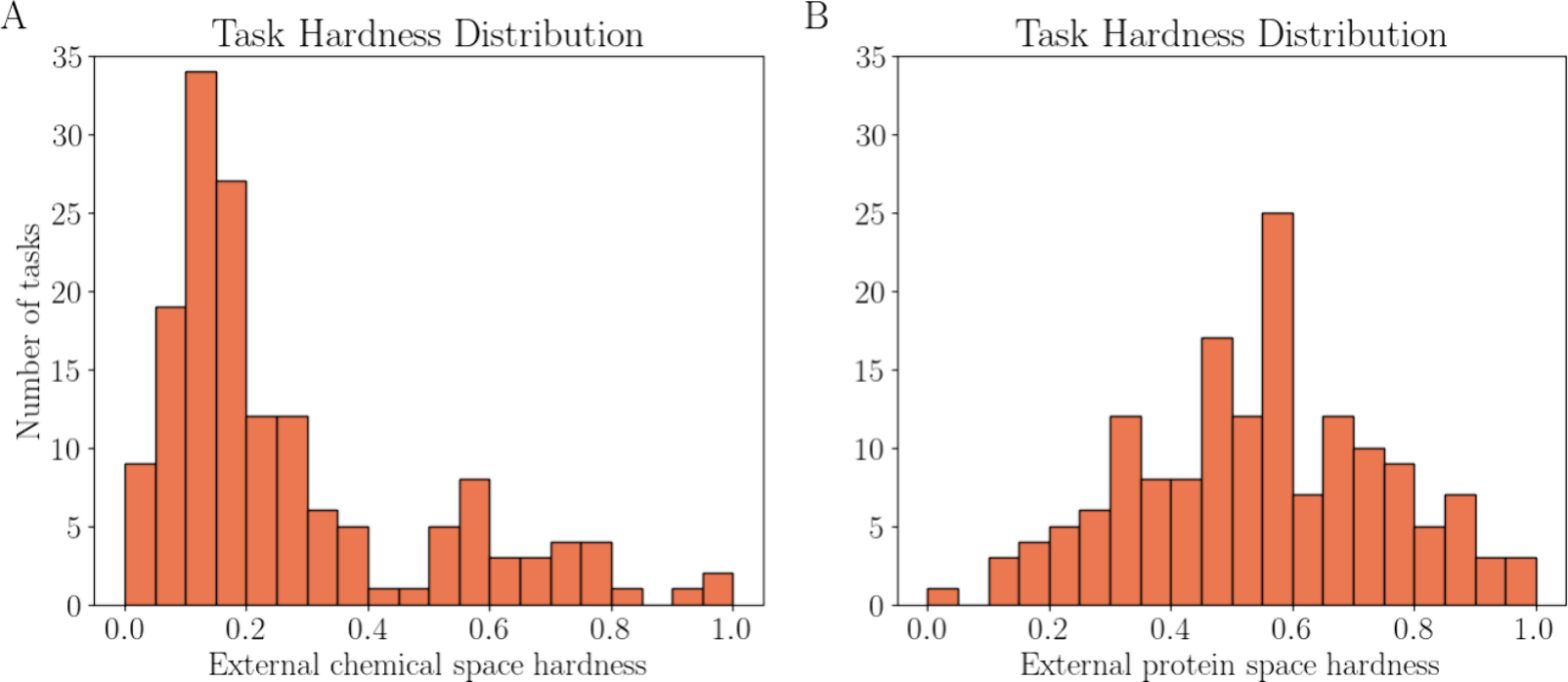
Histograms visualizing the distribution of (normalized) external hardness values for the 157 test tasks included in the FS-Mol benchmark data set. (A) EXT_CHEM derived from GIN-supervised infomax representations of the small molecules. (B) EXT_PROT derived from the ESM2-t33–650 M model. The number of nearest-neighbor training tasks (*k*) for calculating the hardness from the distance matrix is 10.

Figure 6 shows the distribution of the EXT_CHEM and EXT_PROT for the 157 test tasks of the FS-Mol benchmark set by using the default modeling setup. The key observation from the plots in Figure 6 was that the hardness values have a sufficient dynamic range (meaning the values are not concentrated within a narrow range) to be discriminative. The discriminative power is essential for using hardness as a predictor of model performance.

### Sensitivity of the Task Hardness Components on Molecular and Protein Representations

**Figure 7:**
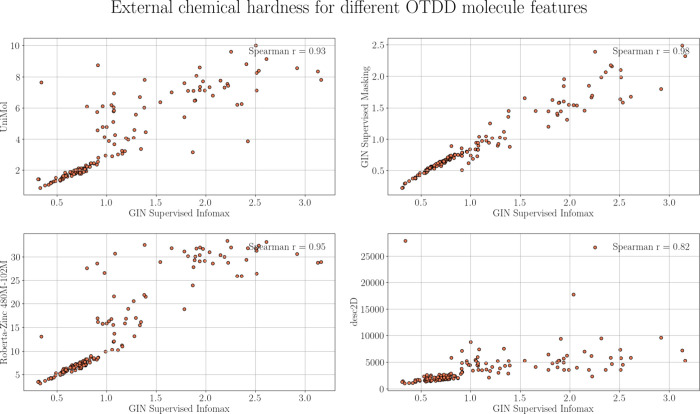
Spearman’s r between EXT_CHEM measures based on different molecule representations. The number of nearest neighbors (*k*; training tasks) for calculating the hardness from the distance matrix is 10.

To determine the sensitivity of the EXT_CHEM to the small-molecule representations, we calculated Spearman’s rank correlation coefficient r for EXT_CHEM values computed with GIN supervised infomax (which we use as the default representation of molecular structures) vs all other explored descriptors (i.e., UniMol, GIN Supervised Masking, Roberta-Zinc-480M-102M, and desc2D; see Methods for details). As shown in Figure 7, all combinations yielded Spearman’s *r* > 0.82. The correlations were stronger for representations from the same modality (e.g., graph representations in GIN infomax and GIN masking), (Spearman’s *r* up to 0.98), whereas for representations from distinct modalities (e.g., graph representations vs SMILES or 3D point-cloud representations), the correlations were weaker (Spearman’s *r* up to 0.95). Together, these results indicate that EXT_CHEM is robust to the small-molecule representation.

**Figure 8:**
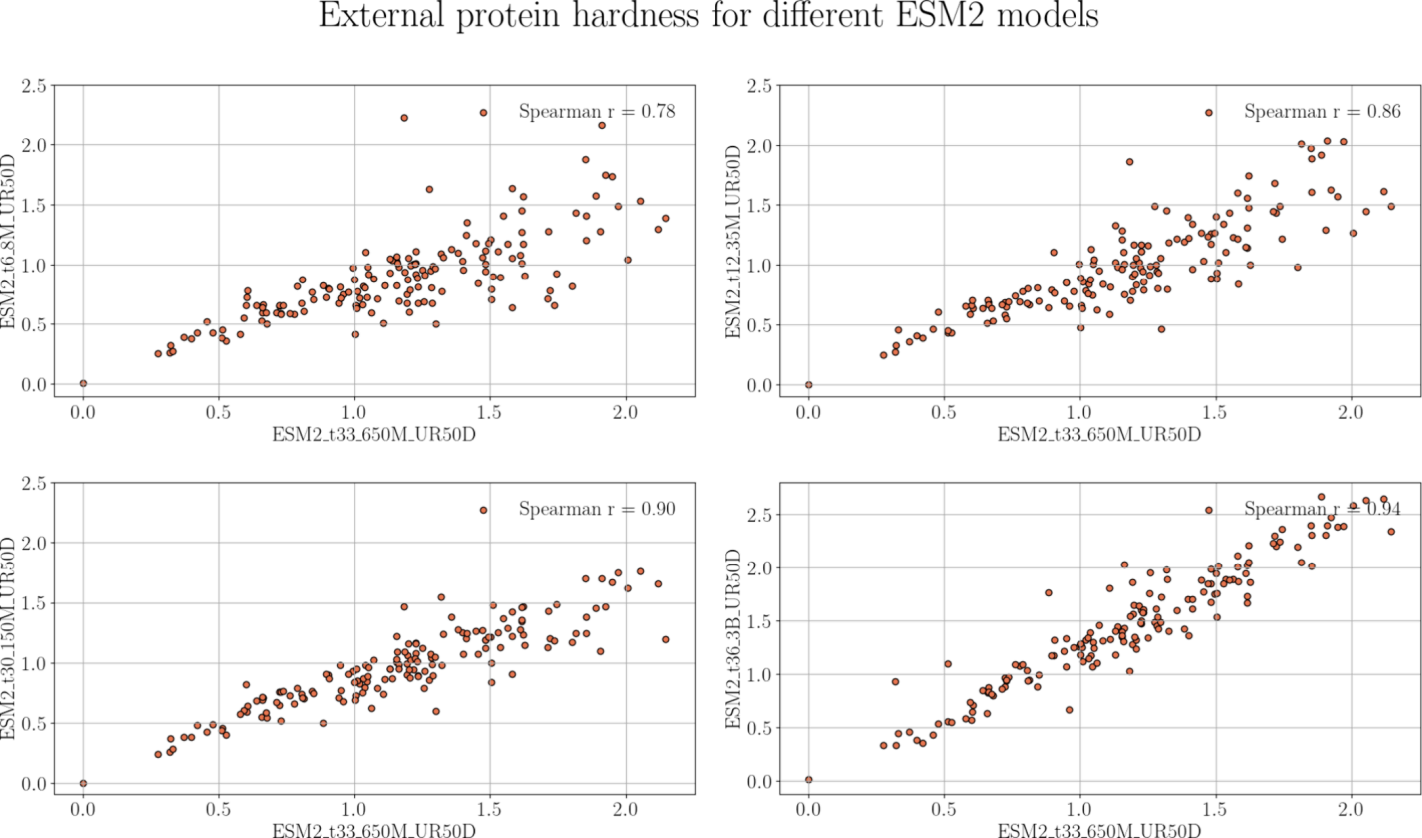
Spearman’s *r* between EXT_PROT measures based on different protein representations. The number of nearest neighbors training data sets (*k*) for calculating the hardness from the distance matrix is 10.

To quantify the sensitivity of the EXT_PROT to protein representations, we again calculated Spearman’s r between the default protein representation, ESM2-t33–650 M (a transformer pretrained with the masked language modeling task), and all other explored descriptors (ESM2-t6–8M, ESM2-t12–35M, ESM2-t30–150 M and ESM2-t36–3B; see Methods for details). As shown in Figure 8, all combinations yielded Spearman’s *r* > 0.86, with one exception. The exception is the comparison of ESM2-t33–650 M with ESM2-t6–8M, a small transformer model with six layers. The Spearman’s r was just 0.78 for this combination, likely because the number of layers and parameters for this combination and, consequently, the generated representations were also very different. Overall, methods based on a similar number of layers and parameters showed higher correlations than those with substantially different numbers of layers and parameters. These results confirm the robustness of the EXT_PROT metric, concerning protein representations.

### Sensitivity of the External Task Hardness Components to the Number of Nearest Source Tasks

To determine the sensitivity of the EXT_CHEM component and the EXT_PROT component to the number of nearest source tasks (*k*), we calculated the Pearson’s *r* for the individual hardness component and the prototypical network performance for different values of *k*. Intuitively, low values of *k* should increase the sensitivity of the task hardness measure to outliers. For example, if *k* = 1 and there is just a single source task that is highly similar to the target task, then the task hardness would be very low (which is not the desirable behavior, because meta-learning is expected to have the largest benefits over single-task models in cases where several of the source tasks are related to the test task). High *k*-values are also expected to diminish the hardness metric’s discriminatory power.

**Figure 9:**
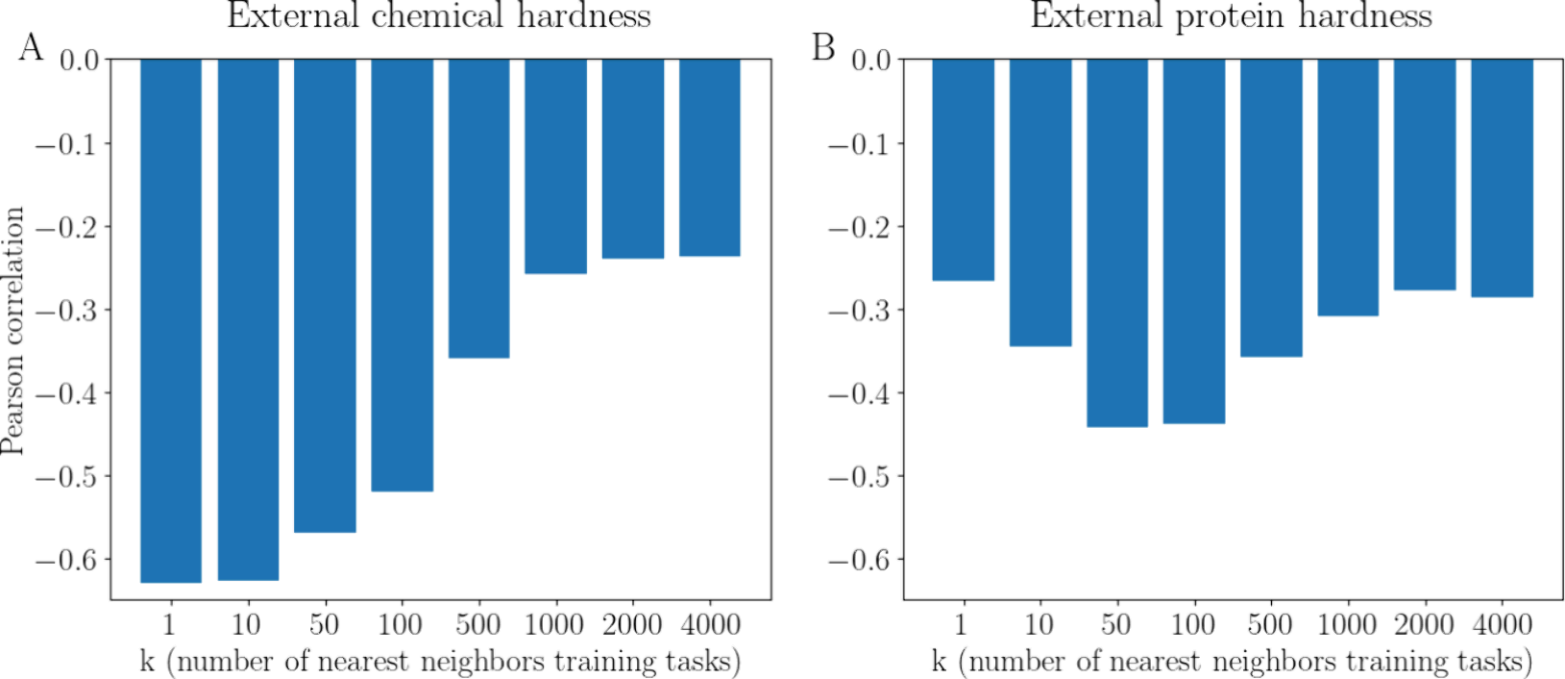
Correlation of (A) EXT_CHEM and (B) EXT_PROT with the performance (ROC-AUC) of the prototypical network as a function of *k* (i.e., the number of nearest neighbor source tasks considered by the hardness components; weighted average used for computing the EXT_CHEM; average used for computing the EXT_PROT). Small molecules represented with GIN supervised infomax; proteins represented with ESM2_t33_650M.

As shown in Figure 9A, low values of *k*, specifically *k* < 100, led to high absolute Pearson’s r between the EXT_CHEM component and the prototypical network performance (Pearson’s *r* = −0.64, −0.63, −0.57 for *k* = 1, 10, 50, respectively). For higher values of *k*, the absolute correlation decreased (Pearson’s *r* = −0.26 for *k* = 1000)

As shown in Figure 9B, low values of *k*, specifically *k* = 1, led to low absolute Pearson’s *r* (Pearson’s *r* = −0.28 for *k* = 1). For *k* > 500, the correlation for EXT_PROT decreased. The highest absolute correlation between the EXT_PROT and the prototypical network performance was observed with 10 < *k* < 100 (i.e., *k* close to 1% of the training tasks).

Most analyses presented in this work are based on using *k* = 10. Results produced with *k* = 50 are available in the Supporting Information.

### Relation between the Task Hardness Components

**Figure 10:**
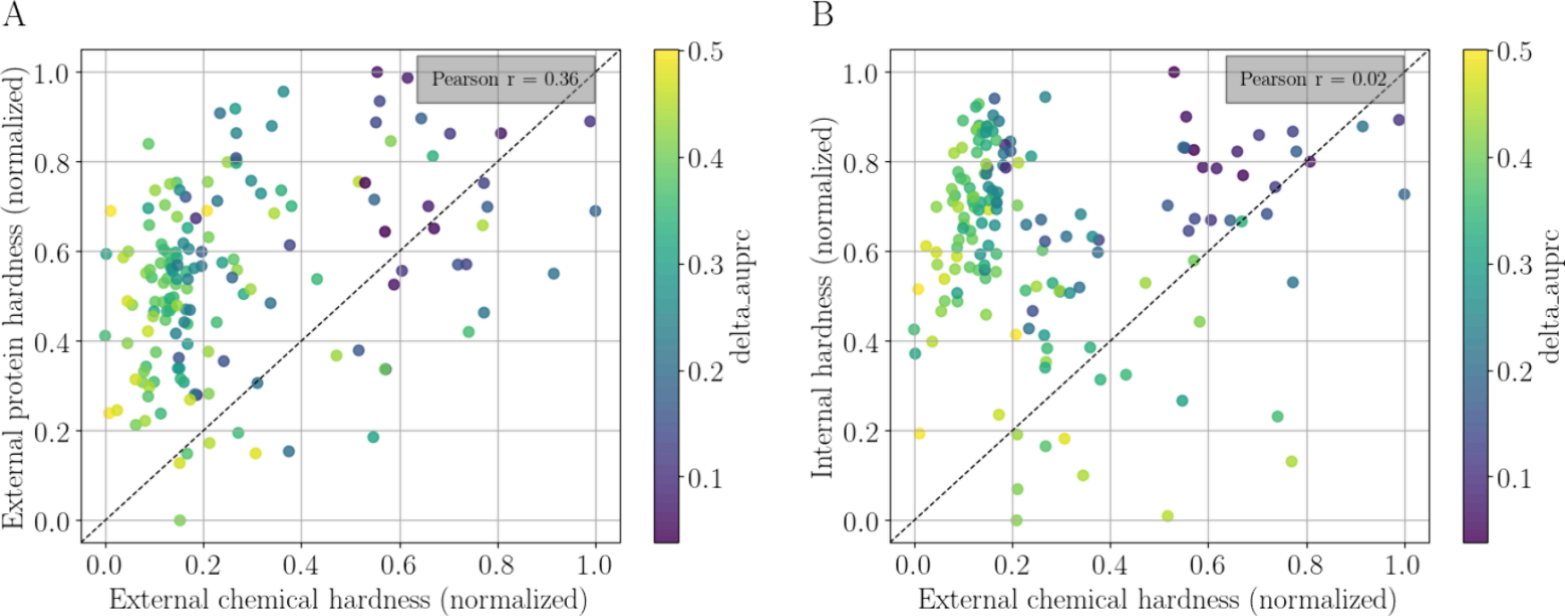
Correlation of EXT_CHEM versus (A) EXT_PROT and (B) INT_CHEM for each of the 157 test tasks. Small molecules represented with GIN supervised infomax; proteins represented with ESM2_t33_650M; number of nearest neighbor source tasks considered by the hardness components *k* = 10, with weighted average used for computing the EXT_CHEM and average used for computing the EXT_PROT; INT_CHEM measured with a random forest model trained on 16 randomly selected training samples.

It is important to know the relation between the individual task hardness components, e.g., to understand whether they capture similar or distinct information. Figure 10A shows the correlation between the EXT_CHEM and EXT_PROT. Each point (task) is colored based on the performance gain of meta-learning (i.e., the prototypical network) over the random forest classifier, measured as ΔAUPRC. Darker colors indicate less improvement in performance when applying the prototypical network. Darker points were observed in the upper right part of the plot, indicating hard tasks, according to both hardness measures. Pearson’s *r* between the EXT_CHEM and EXT_PROT was just 0.36, indicating that these measures shared some information. In other words, this result indicates that closer chemical spaces tend to go along with closer protein spaces.

Figure 10B shows no significant correlation between the EXT_CHEM and INT_CHEM (Pearson’s *r* = 0.02). The two metrics were almost orthogonal, meaning they captured different information about the hardness of a task.

**Figure 11:**
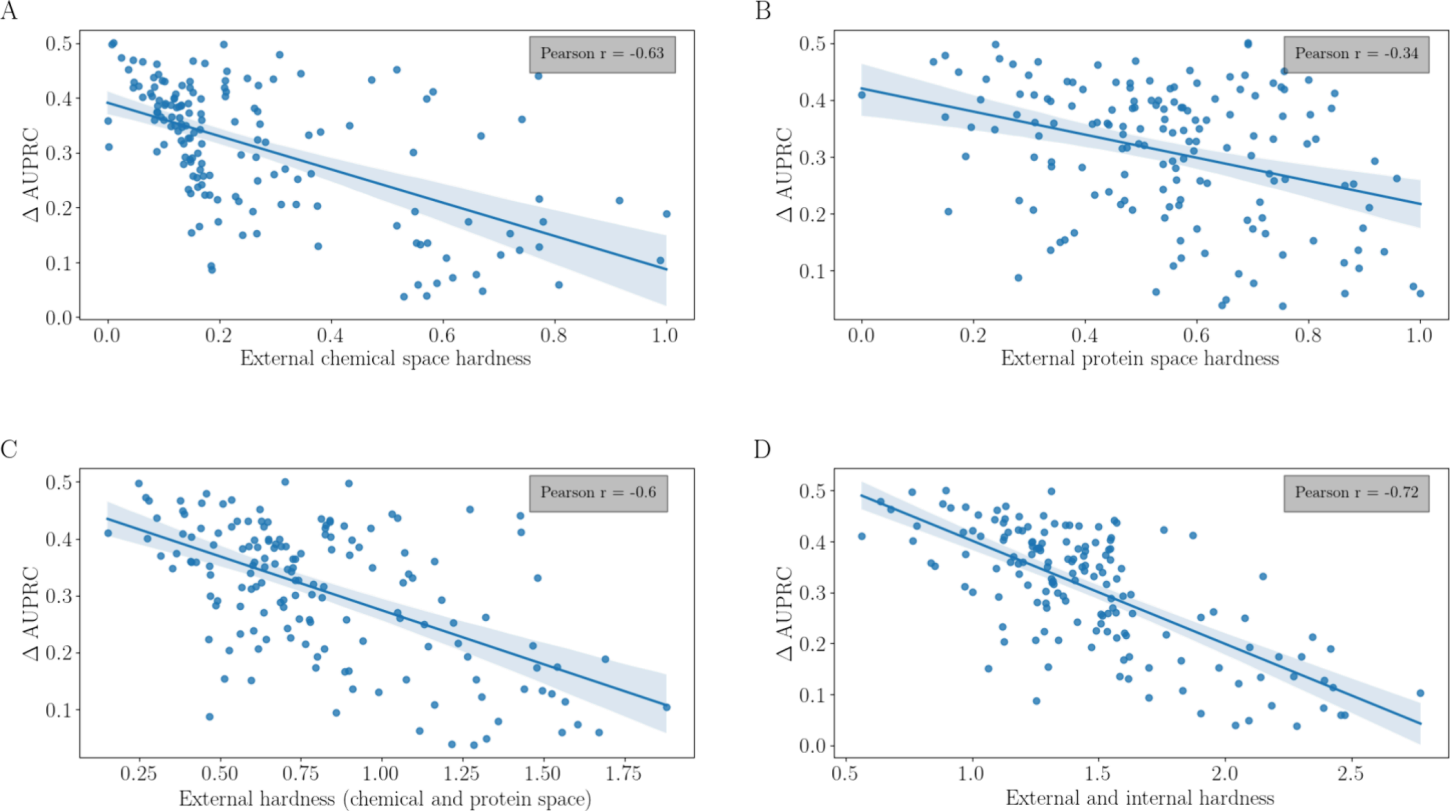
Relationship between the performance improvement (ΔAUPRC) obtained by using the prototypical network (*y*-axis) vs (A) EXT_CHEM, (B) EXT_PROT, (C) EXT_CHEM + EXT_PROT, and (D) EXT_CHEM + EXT_PROT + INT_CHEM. Small molecules represented with GIN supervised infomax; proteins represented with ESM2_t33_650M; number of nearest neighbors (*k*; training tasks) for calculating the hardness from the distance matrix is 10. INT_CHEM was measured with a random forest model trained on 16 randomly selected training samples.

After establishing that the individual task hardness components cover different information, we investigated whether their combination yields a stronger correlation with the performance of the prototypical network. In Figure 11, we visualize the relationship between each hardness concept and the improvement in the performance of the prototypical network (considering a support set size of 128 training samples for each test task; *k* = 10). For EXT_CHEM and the performance of the prototypical network (measured as ΔAUPRC), Pearson’s *r* was −0.63 (Figure 11A). For EXT_PROT and ΔAUPRC, the correlation was weaker (Pearson’s *r* = −0.34; Figure 11B). Adding these two terms together led to Pearson’s *r* = −0.60 (Figure 11C). Finally, adding all three normalized hardness components together improved Pearson’s *r* to −0.72 (Figure 11D).

By exploring all combinations of small-molecule and protein representations, we found combinations reaching Pearson’s *r* values of −0.75 (Table 2). Overall, these results demonstrate that the combined task hardness measure captures a substantial part of the information explaining the difficulty posed by a task to a meta-learning approach. Some additional contributing factors may not yet be fully accounted for.

**Table 2:** Pearson’s *r* for the Final Task Hardness Metric Composed of EXT_CHEM, EXT_PROT and INT_CHEM vs Prototypical Network Performance (ROC-AUC)

	ESM2_t6_8M	ESM2_t12_35M	ESM2_t30_150M	ESM2_t33_650M	ESM2_t36_3B
GIN_Supervised_Infomax	–0.714	–0.723	–0.720	–0.718	–0.697
GIN_Supervised_Masking	–0.734	–0.742	–0.734	–0.735	–0.713
GIN_Supervised_Contextpred	–0.684	–0.701	–0.688	–0.694	–0.668
Roberta-Zinc480M-102M	–0.749	–0.751	–0.748	–0.751	–0.733
Unimol	–0.727	–0.726	–0.723	–0.722	–0.699
Desc2D	–0.585	–0.614	–0.588	–0.584	–0.543
ChemBERTa-77M-MLM	–0.739	–0.741	–0.737	–0.736	–0.717

In Table 2, we reported the Pearson’s *r* for different combinations of small molecule and protein representations. The number of k nearest training tasks for computing hardness from the distance matrix is 10. INT_CHEM for each test task is a random forest classifier (1- ROC-AUC) with 16 samples for training.

### Selection and Training on Relevant Source Tasks for a Particular Target Task

Training a meta-learning approach on a carefully selected set of relevant source tasks rather than the complete data sets could yield competitive models at substantially reduced computational costs. To explore whether the hardness metric presented in this work is useful for selecting relevant source tasks for a specific test task, we determined the performance of a prototypical network trained on the *k* training tasks most closely related, according to the distance and hardness metric, to the test task and compared it to the performance of a prototypical network trained on an equal number of randomly selected training tasks.

On average, for *k* = 500 and a support set size of 128 training samples, we observed a benefit of approximately 6% in ROC-AUC (+0.044) of the rational selection approach (mean ROC-AUC 0.794) over the random selection approach (mean ROC-AUC 0.750). The rational selection approach resulted in higher ROC-AUC values for 80% of the target tasks (125 out of 157).

On average, for *k* = 50 and a support set size of 128 training samples, we observed a benefit of approximately 11% in ROC-AUC (+0.074) of the rational selection approach (mean ROC-AUC 0.772) over the random selection approach (mean ROC-AUC 0.698). The rational selection approach resulted in higher ROC-AUC values for 75% of the target tasks (118 out of 157).

**Figure 12:**
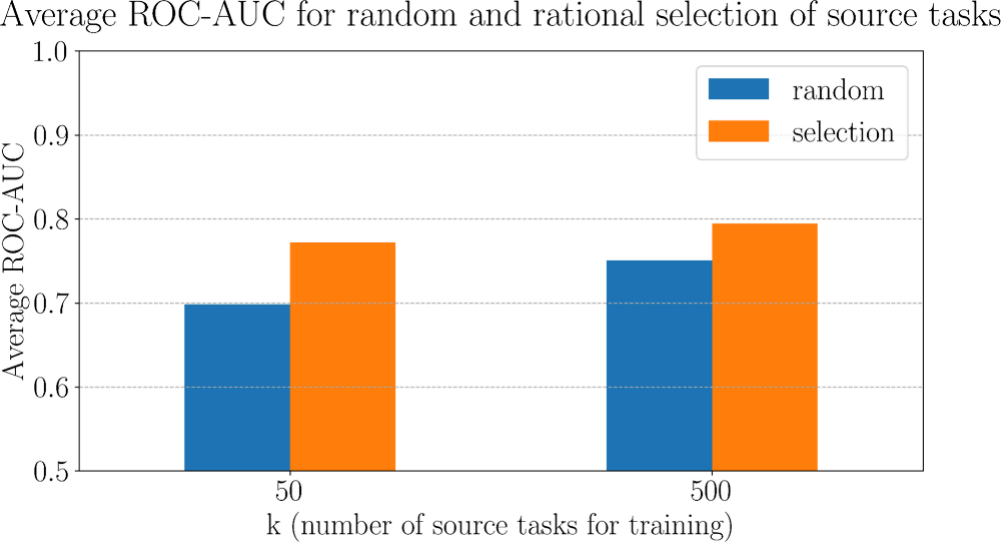
ROC-AUC averages over the 157 target tasks using 50 or 500 source tasks for training the prototypical networks. For the rational selection, small molecules were represented with GIN supervised infomax and proteins with ESM2_t33_650M. The support set size of the prototypical network is 128.

Overall, as *k* decreased, the overall performance of meta-learning tended to also decrease (Figure 12). However, the benefit of the performance of the rational selection approach outweighed that trend. As *k* increased, the overall performance improved and the gap between the performance of rational and random selection narrowed.

### Computational and Time Complexity of Our Proposed Method

All experiments were conducted on a Linux machine equipped with an AMD Ryzen 9 7950X 16-Core Processor, 128 GB of RAM, and one NVIDIA GeForce RTX 4090 GPU. Featurization of the full set of 482,358 small-molecules (SMILES) with featurizers such as GIN supervised infomax and the full set of protein sequences with featurizers such as ESM2_t33_650 M took approximately 5 min in total when running 16 jobs in parallel. Calculation of the Euclidean distance between two matrices (protein representation) was completed within seconds. The most time-consuming is generating the OTDD-based distance matrix for the 4938 source and 157 target tasks. This process took approximately 1 h on the GPU.

## Conclusions

We introduce a new metric that quantifies the hardness (difficulty) of a bioactivity prediction task based on the relatedness of the chemical and bioactivity data available for training knowledge-sharing machine-learning approaches. Using the prototypical network as an example of a meta-learning approach, we show that the new hardness metric correlates well with the prediction success of the prototypical network. We also confirm that the hardness metric is robust when using different molecular and protein representations and parameters.

In practice, the new metric can be used to (i) predict the advantage of knowledge-sharing vs single-task approaches for specific prediction tasks and (ii) select relevant source tasks for optimum performance gain of knowledge-sharing methods.

## Data Availability

All data utilized in this work originates from the FS-Mol data set and is available from https://github.com/microsoft/FS-Mol. The source code of the approach presented in this work is available from https://github.com/HFooladi/THEMAP.

## Supporting Information Available

Further details on the molecule and protein embeddings, the prototypical network architecture, data set statistics, and analysis of the task hardness for various hyperparameters (PDF)
