# Supplementary material for: Quantifying the Hardness of Bioactivity Prediction Tasks for Transfer Learning
Source: J Chem Inf Model. 2024 May 13;64(10):4031–46. doi: 10.1021/acs.jcim.4c00160 (PMC11134514; doi:10.1021/acs.jcim.4c00160)
Supplement: Supplementary file 1 — ci4c00160_si_001.pdf [file ci4c00160_si_001.pdf]

SUPPORTING INFORMATION

Quantifying the hardness of bioactivity prediction tasks for transfer learning

Hosein Fooladi,[1,2,3] Steffen Hirte,[1,3] and Johannes Kirchmair[1,2*]

[1] Department of Pharmaceutical Sciences, Division of Pharmaceutical Chemistry, Faculty of Life Sciences, University of Vienna, Josef-Holaubek-Platz 2, 1090 Vienna, Austria

[2] Christian Doppler Laboratory for Molecular Informatics in the Biosciences, Department for Pharmaceutical Sciences, University of Vienna, 1090 Vienna, Austria

[3] Vienna Doctoral School of Pharmaceutical, Nutritional and Sport Sciences (PhaNuSpo), University of Vienna, 1090 Vienna, Austria

* Corresponding author: johannes.kirchmair@univie.ac.at

Protein Embedding

Protein embeddings were calculated from their amino acid sequences. First, we recovered the target ChEMBL ID from the assay ChEMBL ID. Then, we extracted the UniProt ID for each target ChEMBL ID. Subsequently, we extracted sequences belonging to each Uniprot ID.

The amino acid sequence was used as the input for protein representation learning. The following ESM-2 models[1,2] have been used for learning protein representation.

- ESM2 (esm2_t6_8M_UR50D): This transformer has 6 layers and 8 million trainable parameters. The mean output of the last layer (layer 6) was used as the protein representation. The command that has been used is:

```
python scripts/extract.py esm2_t6_8M_UR50D fsmol_sequences.fasta embeddings_output –repr_layers 6 –include mean –truncation_seq_length 4096
```

  The output of this model is a 320-dimensional array for each protein (amino acid sequence)

- ESM2 (esm2_t12_35M_UR50D): This is a transformer with 12 layers and 35 million trainable parameters. The mean output of the last layer (layer 12) was used as the protein representation. The exact command that has been used is:

```
python scripts/extract.py esm2_t12_35M_UR50D fsmol_sequences.fasta embeddings_output --repr_layers 12 --include mean --truncation_seq_length 4096
```

  The output of this model is a 480-dimensional array for each protein (amino acid sequence)

- ESM2 (esm2_t30_150M_UR50D): This is a transformer with 30 layers and 150 million trainable parameters. The mean output of the last layer (layer 30) was used as the protein representation. The exact command that has been used is:

```
python scripts/extract.py esm2_t30_150M_UR50D fsmol_sequences.fasta embeddings_output –repr_layers 30 –include mean –truncation_seq_length 4096
```

  The output of this model is a 640-dimensional array for each protein (amino acid sequence)

- ESM2 (esm2_t33_650M_UR50D): This is a transformer with 33 layers and 650 million trainable parameters, this outputs a representation for each token (amino acid residue) at each layer. The output of the last layer (layer 33) was used in this study. Also, to determine the representation of the whole protein (not just amino acid residues), the mean representation of each token (amino acid residue) was used. The exact command that has been used is:

```
python scripts/extract.py esm2_t33_650M_UR50D fsmol_sequences.fasta embeddings_output --repr_layers 33 --include mean --truncation_seq_length 4096
```

  The output of this model is a 1280-dimensional array for each protein (amino acid sequence)

- ESM2 (esm2_t36_3B_UR50D): This is a transformer with 36 layers and 3 billion trainable parameters. The mean output of the last layer (layer 36) was used as the protein representation. The exact command that has been used is:

```
python scripts/extract.py esm2_t36_3B_UR50D fsmol_sequences.fasta embeddings_output --repr_layers 36 --include mean --truncation_seq_length 4096
```

  The output of this model is a 2560-dimensional array for each protein (amino acid sequence)

Molecule featurization

Table S1 lists the methods (featurizers) used for calculating the molecular representations explored in this study.

Molfeat

Software version: Molfeat commit hash 4390f9f from authors' public code repository: https://github.com/datamol-io/molfeat

The input to the featurizer is a SMILES or list of SMILES, and the output will be the features for each specific SMILES.

Unimol

Software version: Uni-Mol commit hash b6427ce from authors' public code repository: https://github.com/dptech-corp/Uni-Mol

The input to the featurizer is a SMILES or list of SMILES, and the output will be the features for each specific smiles. The output is a 512-dimensional array.

Distance module

Optimal transport dataset distance (OTDD)

For determining the distance between chemical space (molecule-label pairs), the optimal transport dataset distance was used.[3]

Software version: otdd commit hash 72f1b22 from authors' public code repository https://github.com/microsoft/otdd

The algorithm takes a pair of inputs (x, y), where x represents a molecule, and y is a bioactivity label (a binary value indicating the molecule's activity on a target). Various features or representations obtained from featurizers are utilized for the molecular representation (x).

```
dist = DatasetDistance(loaders_src, loaders_tgt,
                       inner_ot_method = 'exact',
                       debiased_loss = True,
                       p = 2, entreg = 1e-1,
                       device='cuda')

d = dist.distance(maxsamples = 1000)
```

All parameters are set to their default values as used in the original repository. Additionally, 'max_samples' has been set to 1000, although this does not impact our results significantly, as the majority of our dataset comprises fewer than 1000 samples. Consequently, the outcome is not sensitive to this parameter.

Prototypical Network

To validate the relevance of our proposed distance and hardness module, we compared the performance of a prototypical network[4] with the assigned hardness for each task. We utilized the FS-Mol implementation of the prototypical network, available at https://github.com/microsoft/FS-Mol. Specifically, the benchmarking study employed the following architecture and features for the prototypical network:

Features and Architecture:

- Fully connected layer on top of features derived from a graph neural network (GNN) and Extended Connectivity Fingerprint (ECFP).

GNN Characteristics:

- Type: Principled Neighborhood Aggregation (PNA).
- Number of heads: 4
- Number of layers: 10
- Per head dimensions: 32
- Hidden dimensions: 128
- Distance function between samples and prototypes: Mahalanobis distance.

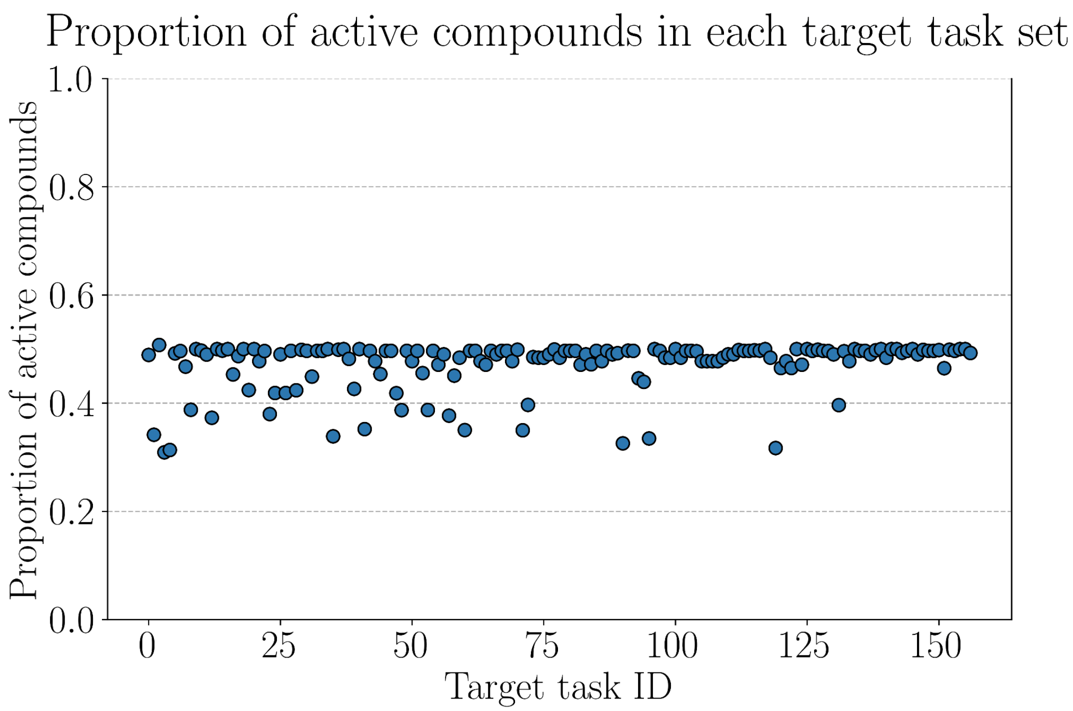

Figure S1: Proportion of active compounds in each target task set. Most target task sets comprise a comparable number of active and inactive compounds.

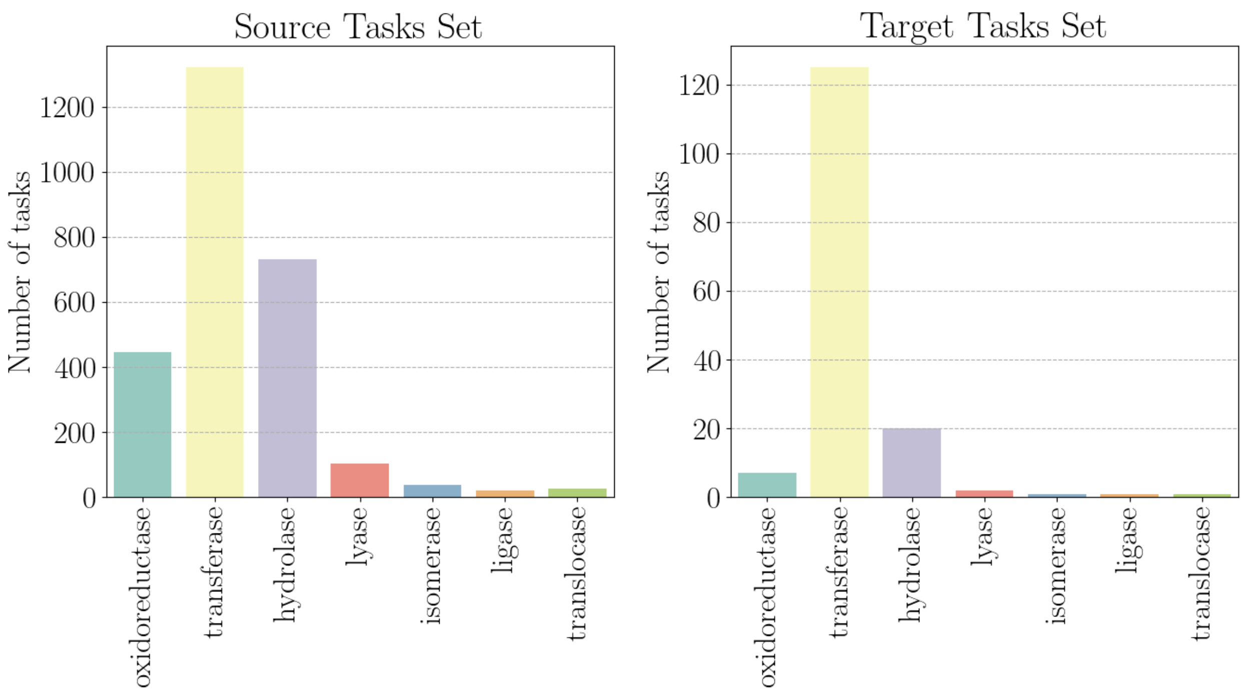

Figure S2: Representation of enzyme types among the single protein assay source tasks and target tasks within the FS-Mol data set. Transferases dominate both data sets, followed by hydrolases and oxidoreductases. Within the source tasks, some tasks have more than one assigned protein family (7%) or no specification of a protein family (33%). These are not reported in this figure.

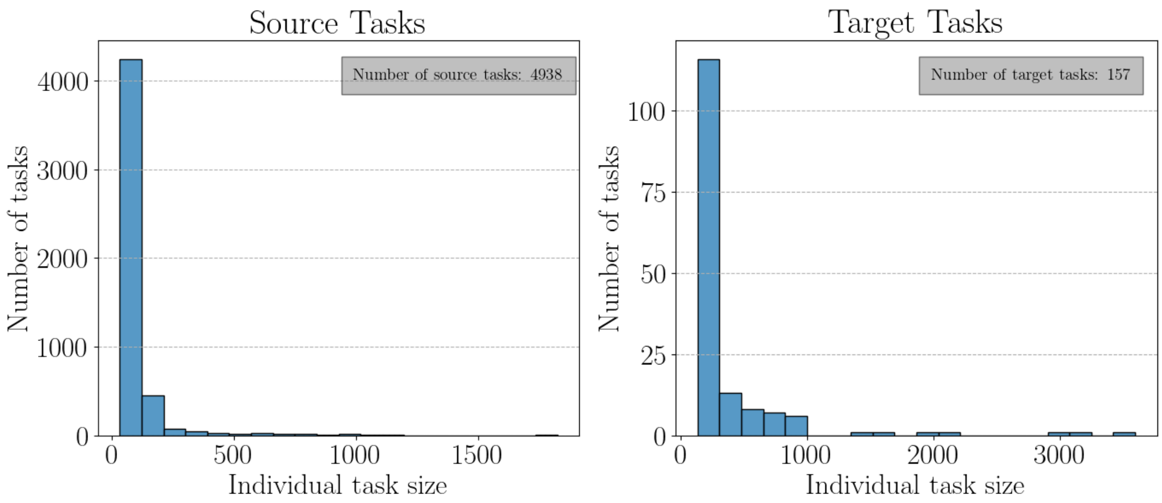

Figure S3: Number of molecules representing the individual source and target tasks within the FS-Mol data set.

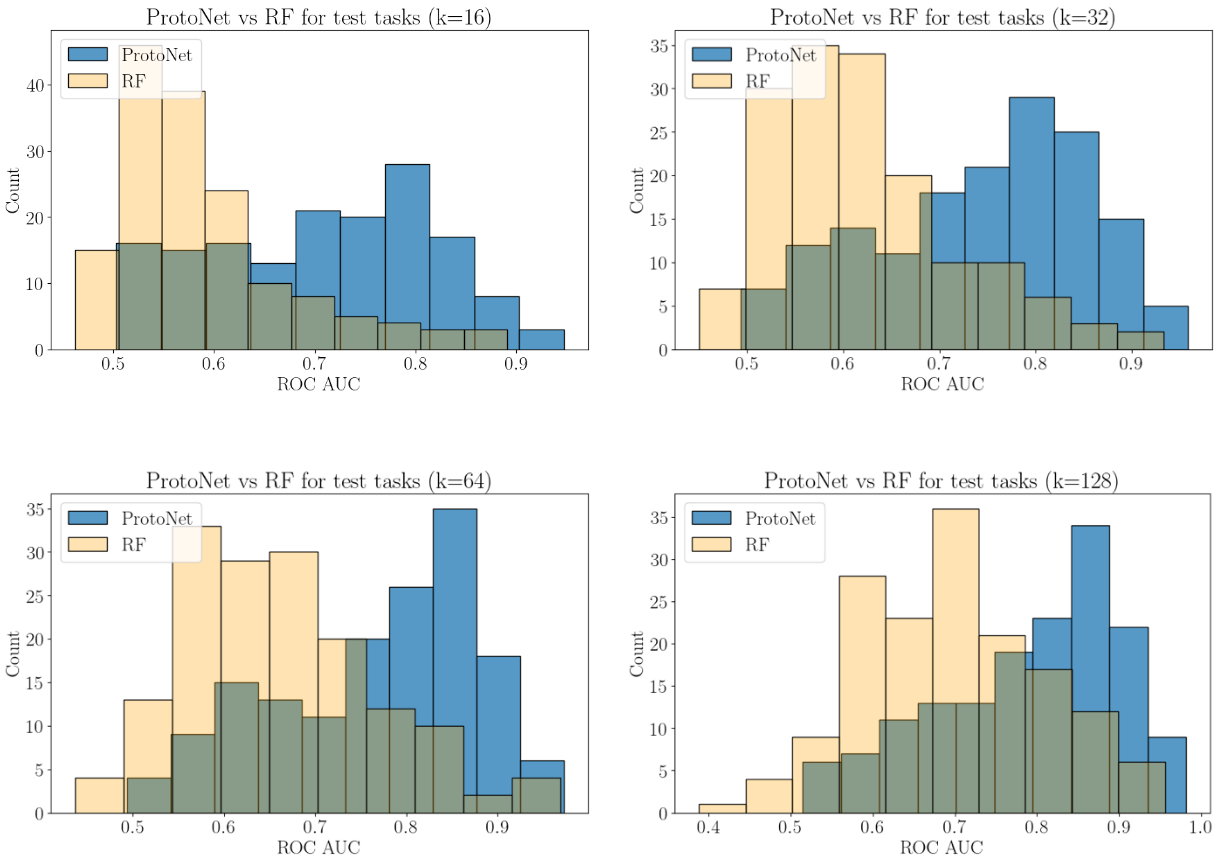

Figure S4: Meta-learning, in particular the prototypical network, achieved better performance (measured as ROC-AUC) than random forest (a single-task method) for all values of k (number of training data points). Increasing the number of training samples improved the performance of both methods but also shrunk the performance gap between the two methods (the more training data is available, the less improvement would be seen from a prototypical network compared to a single-task approach).

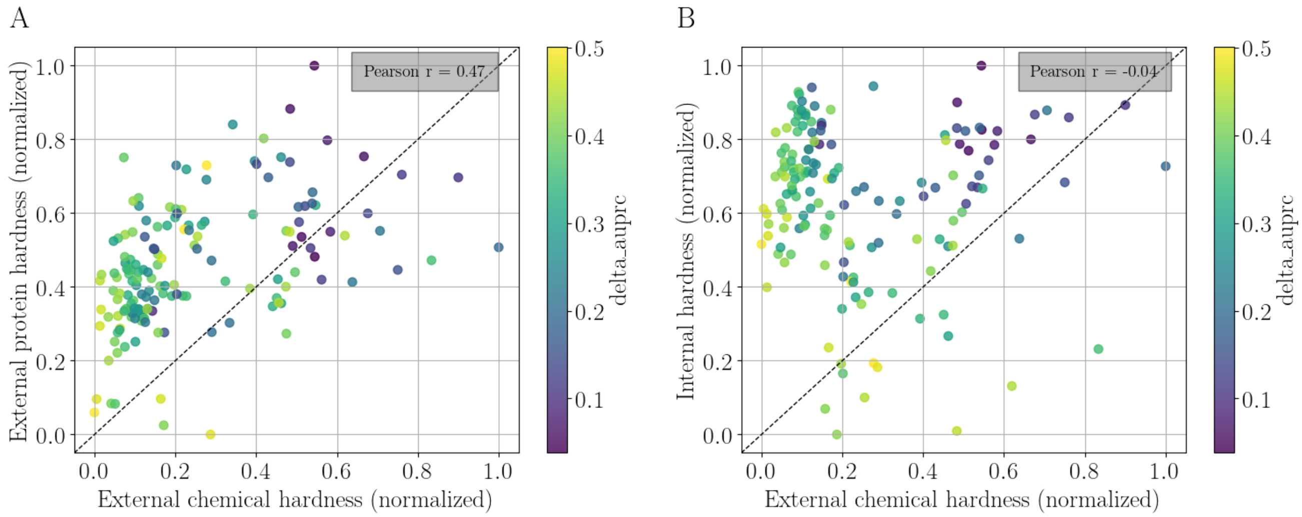

Figure S5: Correlation of EXT_CHEM vs (A) EXT_PROT and (B) INT_CHEM for each of the 157 test tasks. Small molecules represented with GIN supervised infomax; proteins represented with ESM2_t33_650M; The number of nearest neighbors (k; training tasks) for calculating the hardness from the distance matrix is 50, with the weighted average used for computing the EXT_CHEM and average used for computing the EXT_PROT; INT_CHEM measured with a random forest model trained on 16 randomly selected training samples.

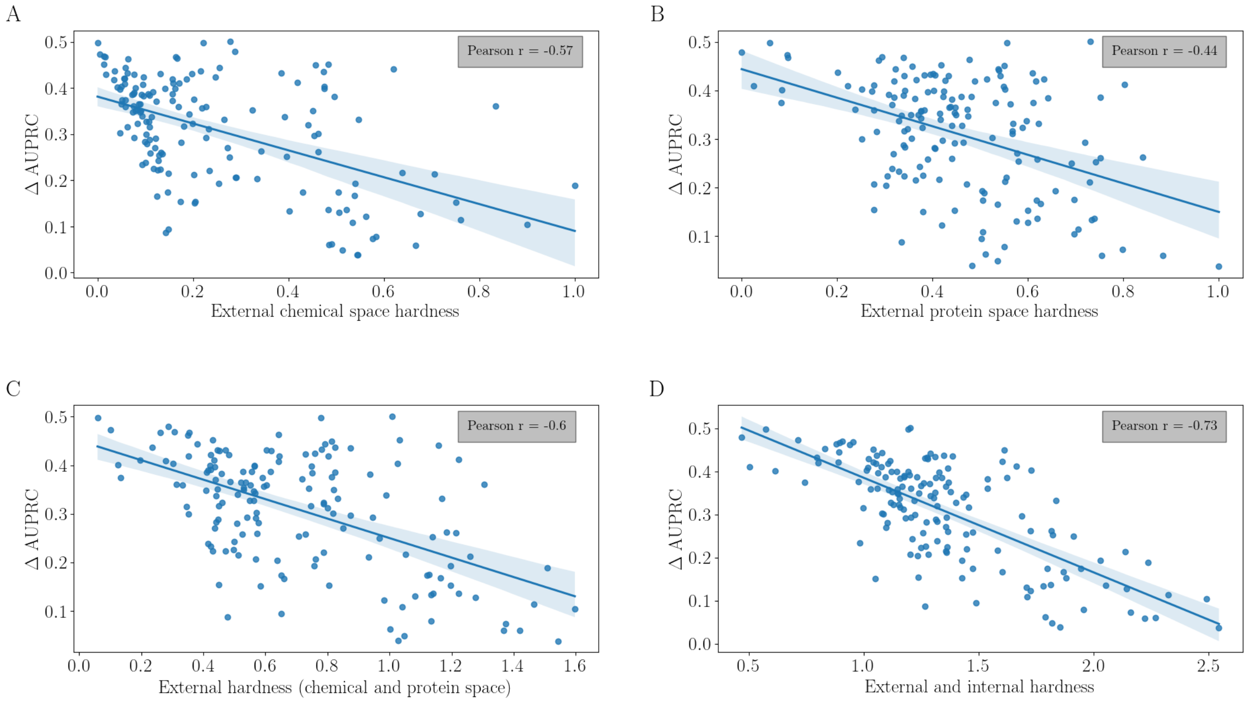

Figure S6: Relationship between the performance improvement (ΔAUPRC) obtained by using the prototypical network (y-axis) vs (A) EXT_CHEM, (B) EXT_PROT, (C) EXT_CHEM + EXT_PROT, and (D) EXT_CHEM + EXT_PROT + INT_CHEM. The number of nearest neighbors (k; training tasks) for calculating the hardness from the distance matrix is 50.

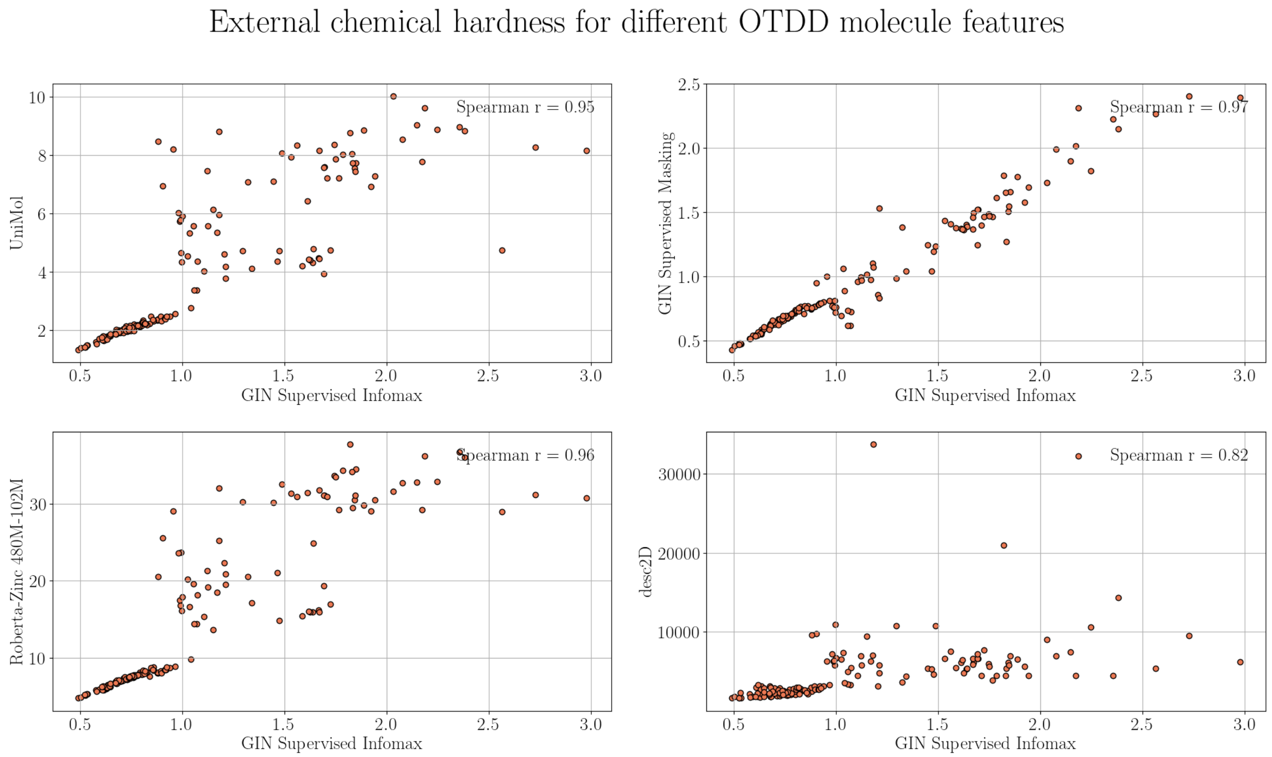

Figure S7: Spearman's r between EXT_CHEM measures based on different molecule representations. The number of nearest neighbors (k; training tasks) for calculating the hardness from the distance matrix is 50.

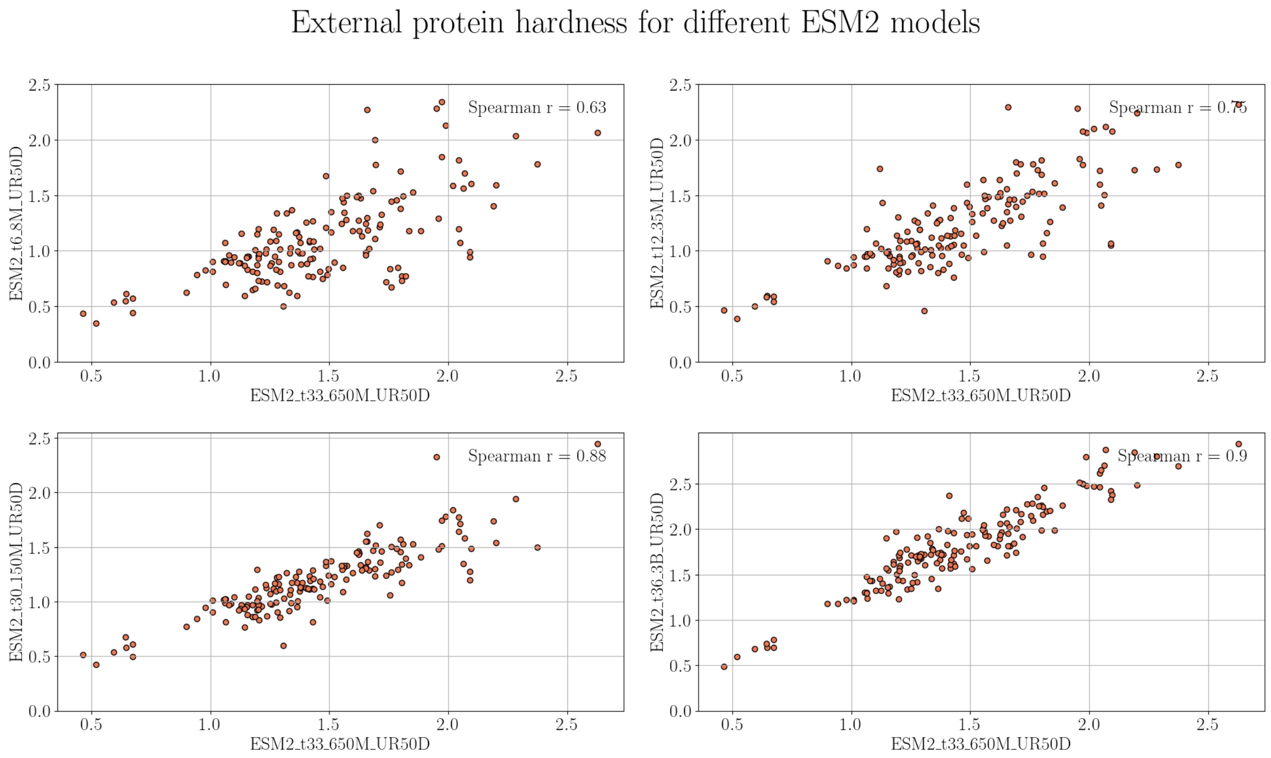

Figure S8: Spearman's r between EXT_PROT measures based on different protein representations. The number of nearest neighbors (k; training tasks) for calculating the hardness from the distance matrix is 50.

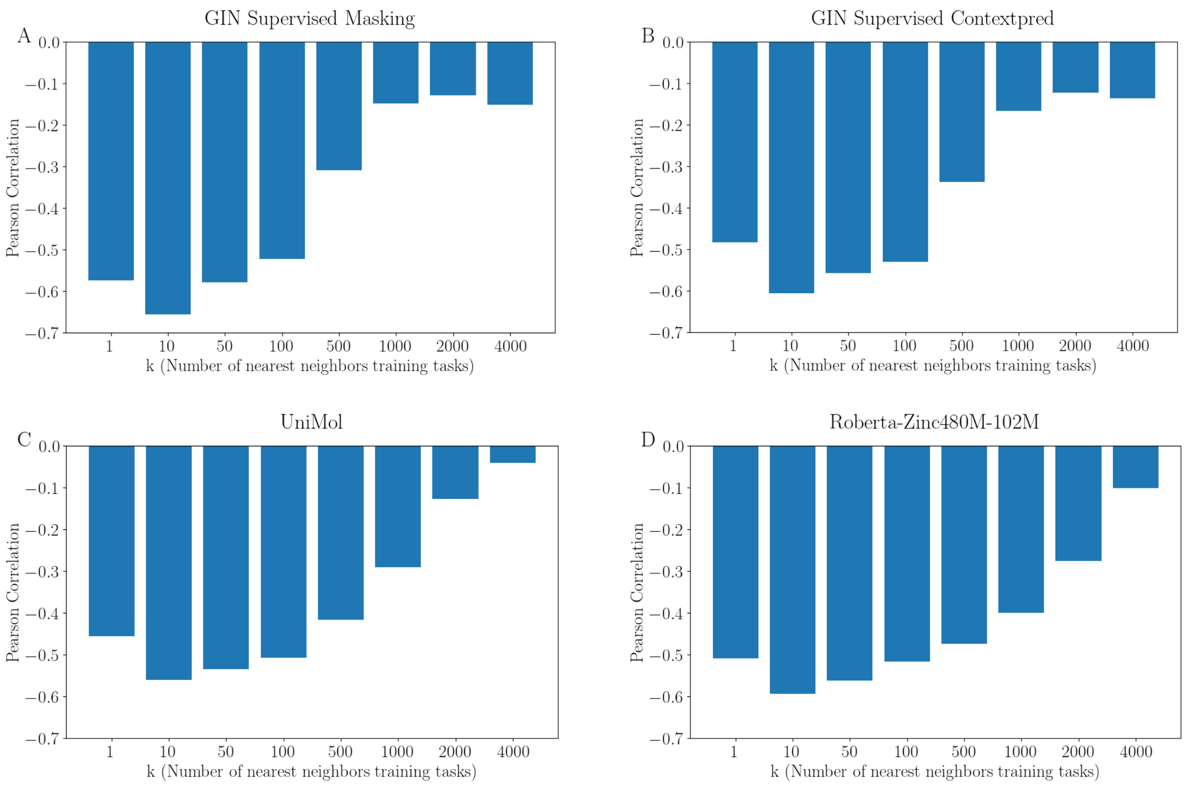

Figure S9: Pearson's r of the EXT_CHEM with the performance (ROC-AUC) of the prototypical network as a function of k (i.e., the number of nearest neighbor source tasks considered by the hardness components; weighted average used for computing the EXT_CHEM) (A) GIN supervised masking, (B) GIN supervised contextpred, (C), UniMol, and (D) Roberta-Zinc 480M-102M. This figure shows that correlation is sensitive to this parameter, and based on this plot, around 0.1-1% of source tasks can be an optimum number for k.

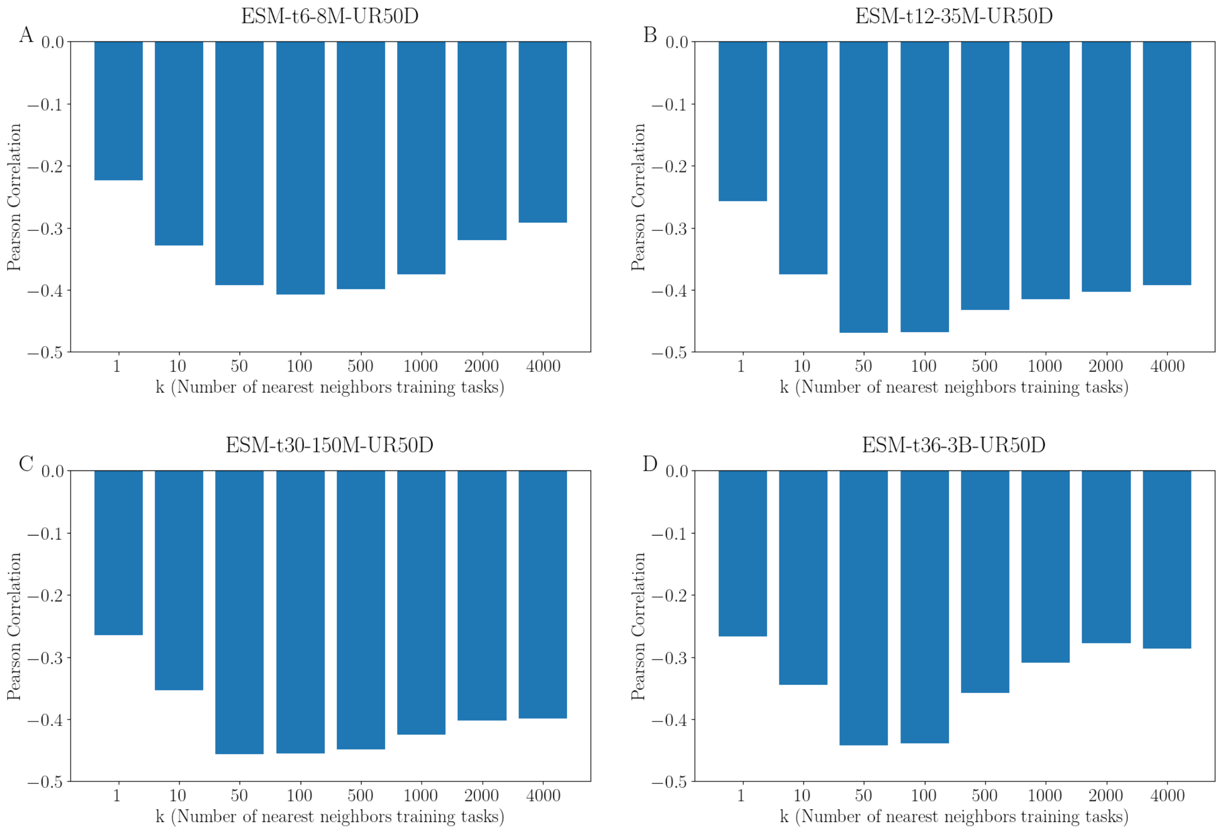

Figure S10: Pearson's r of the EXT_PROT with the performance (ROC-AUC) of the prototypical network as a function of k (i.e., the number of nearest neighbor source tasks considered by the hardness components; average used for computing the EXT_PROT) (A) ESM2_t6_8M model, (B) ESM2_t12_35M model, (C), ESM2_t30_150M model, and (D) ESM2_t36_3B model. This figure shows that correlation is sensitive to this parameter, and based on this plot, around 0.1-1% of source tasks can be an optimum number for k.

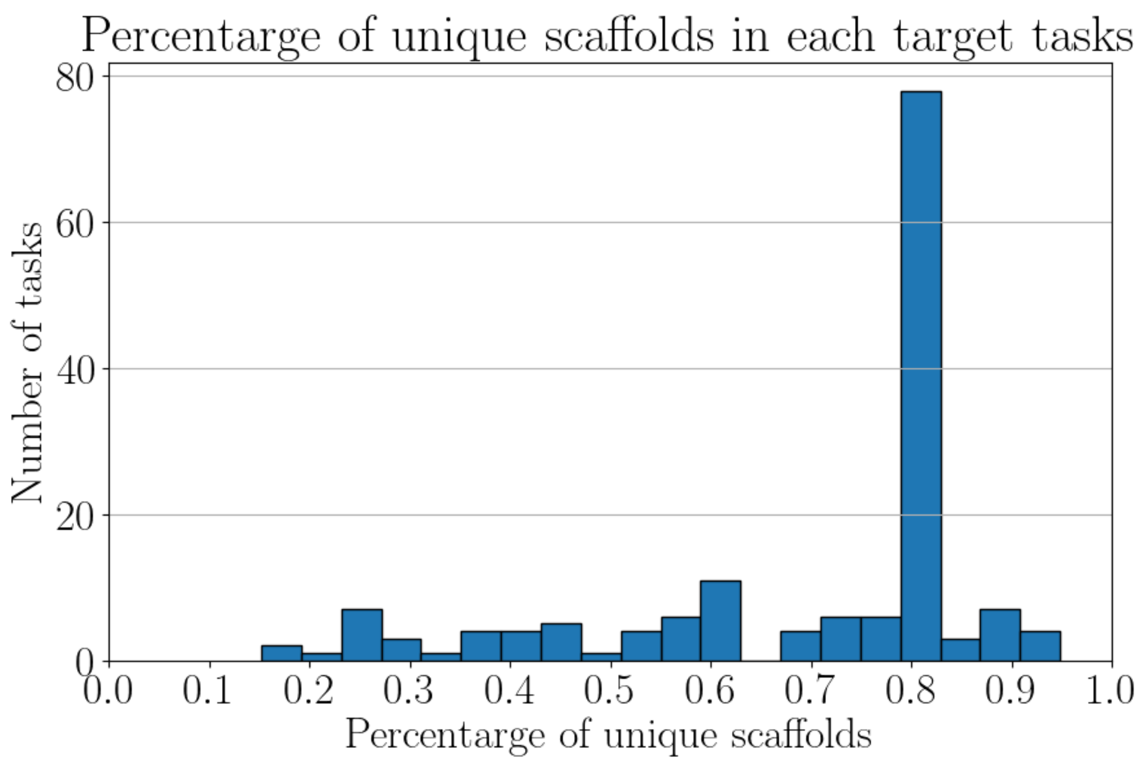

Figure S11: Percentage of unique scaffolds (Bemis-Murcko scaffolds) in each target task set.

Table S1: List of molecular featurizers.

Featurizer	Input	Output
2D physio-chemical descriptors from RDKit[5]	SMILES	215-dimensional array
ChemBERTa-77M-MTR[6]	SMILES	384-dimensional array
ChemBERTa-77M-MLM[6]	SMILES	384-dimensional array
Unimol[7]	SMILES	512-dimensional array
Gin_supervised_contextpred[8]	SMILES	300-dimensional array
Gin_supervised_edgepred[8]	SMILES	300-dimensional array
Gin_supervised_masking[8]	SMILES	300-dimensional array
Gin_supervised_infomax[8]	SMILES	300-dimensional array
Roberta-Zinc480M-102M	SMILES	768-dimensional array

Table S2: Pearson's r for the final task hardness metric composed of EXT_CHEM, EXT_PROT, and INT_CHEM vs prototypical network performance (ROC-AUC). The number of nearest neighbors (k; training tasks) for calculating the hardness from the distance matrix is 50. Internal hardness for each test task is random forest ROC-AUC with 16 data points (samples) for training.

	**ESM2_ t6_8M**	**ESM2_ t12_35M**	**ESM2_ t30_150M**	**ESM2_ t33_650M**	**ESM2_ t36_3B**
GIN_ Supervised_ Infomax	-0.702	-0.738	-0.738	-0.733	-0.711
GIN_ Supervised_ Masking	-0.706	-0.739	-0.738	-0.736	-0.716
GIN_ Supervised_ Contextpred	-0.695	-0.733	-0.727	-0.727	-0.705
Roberta-Zinc480M-102M	-0.724	-0.740	**-0.751**	-0.749	-0.734
Unimol	-0.713	-0.727	-0.742	-0.739	-0.720
Desc2D	-0.601	-0.652	-0.636	-0.630	-0.587
ChemBERa-77M-MLM	-0.722	-0.744	-0.749	-0.745	-0.725

Table S3: Pearson's r for the final task hardness metric composed of EXT_CHEM, EXT_PROT, and INT_CHEM vs prototypical network performance (ROC-AUC). The number of nearest neighbors (k; training tasks) for calculating the hardness from the distance matrix is 10. Internal hardness for each test task is random forest ROC-AUC with 64 data points (samples) for training.

	**ESM2_ t6_8M**	**ESM2_ t12_35M**	**ESM2_ t30_150M**	**ESM2_ t33_650M**	**ESM2_ t36_3B**
GIN_ Supervised_ Infomax	-0.744	-0.748	-0.745	-0.742	-0.722
GIN_ Supervised_ Masking	-0.768	**-0.771**	-0.763	-0.763	-0.742
GIN_ Supervised_ Contextpred	-0.768	-0.738	-0.725	-0.730	-0.705
Roberta-Zinc480M-102M	-0.765	-0.765	-0.761	-0.763	-0.747
Unimol	-0.749	-0.744	-0.741	-0.738	-0.717
Desc2D	-0.642	-0.661	-0.635	-0.626	-0.586
ChemBERa-77M-MLM	-0.745	-0.766	-0.762	-0.760	-0.741

Table S4: Pearson r for INT_CHEM with different splitting strategies vs prototypical network performance (ROC-AUC).

	**RF (k=16)**	**RF (k=32)**	**RF (k=64)**	**kNN (k=16)**	**kNN (k=32)**	**kNN (k=64)**
Random splitting	-0.369	-0.416	-0.520	-0.381	-0.492	-0.529
Stratified random splitting	-0.429	-0.481	-0.529	-0.454	-0.502	-0.523
Scaffold splitting	-0.330	-0.398	-0.503	-0.379	-0.481	-0.514

References

(1) Lin, Z.; Akin, H.; Rao, R.; Hie, B.; Zhu, Z.; Lu, W.; Smetanin, N.; Verkuil, R.; Kabeli, O.; Shmueli, Y.; Dos Santos Costa, A.; Fazel-Zarandi, M.; Sercu, T.; Candido, S.; Rives, A. Evolutionary-Scale Prediction of Atomic-Level Protein Structure with a Language Model. *Science* **2023**, *379*, 1123–1130. https://doi.org/10.1126/science.ade2574.

(2) Rives, A.; Meier, J.; Sercu, T.; Goyal, S.; Lin, Z.; Liu, J.; Guo, D.; Ott, M.; Zitnick, C. L.; Ma, J.; Fergus, R. Biological Structure and Function Emerge from Scaling Unsupervised Learning to 250 Million Protein Sequences. *Proc. Natl. Acad. Sci.* **2021**, *118*, e2016239118. https://doi.org/10.1073/pnas.2016239118.

(3) Alvarez-Melis, D.; Fusi, N. Geometric Dataset Distances via Optimal Transport. arXiv February 7, 2020. https://doi.org/10.48550/arXiv.2002.02923.

(4) Snell, J.; Swersky, K.; Zemel, R. S. Prototypical Networks for Few-Shot Learning. arXiv June 19, 2017. http://arxiv.org/abs/1703.05175 (accessed 2023-08-30).

(5) Landrum, G.; Tosco, P.; Kelley, B.; Ric; Cosgrove, D.; Sriniker; Gedeck; Vianello, R.; NadineSchneider; Kawashima, E.; N, D.; Jones, G.; Dalke, A.; Cole, B.; Swain, M.; Turk, S.; AlexanderSavelyev; Vaucher, A.; Wójcikowski, M.; Ichiru Take; Probst, D.; Ujihara, K.; Scalfani, V. F.; Godin, G.; Lehtivarjo, J.; Pahl, A.; Walker, R.; Francois Berenger; Jasondbiggs; Strets123. Rdkit/Rdkit: 2023_03_2 (Q1 2023) Release, 2023. https://doi.org/10.5281/ZENODO.8053810.

(6) Ahmad, W.; Simon, E.; Chithrananda, S.; Grand, G.; Ramsundar, B. ChemBERTa-2: Towards Chemical Foundation Models. **2022**. https://doi.org/10.48550/ARXIV.2209.01712.

(7) Zhou, G.; Gao, Z.; Ding, Q.; Zheng, H.; Xu, H.; Wei, Z.; Zhang, L.; Ke, G. *Uni-Mol: A Universal 3D Molecular Representation Learning Framework*; preprint; Chemistry, 2022. https://doi.org/10.26434/chemrxiv-2022-jjm0j.

(8) Hu, W.; Liu, B.; Gomes, J.; Zitnik, M.; Liang, P.; Pande, V.; Leskovec, J. Strategies for Pre-Training Graph Neural Networks. **2019**. https://doi.org/10.48550/ARXIV.1905.12265.
